# Lamivudine modulates the expression of neurological impairment-related genes and LINE-1 retrotransposons in brain tissues of a Down syndrome mouse model

**DOI:** 10.3389/fnagi.2024.1386944

**Published:** 2024-07-19

**Authors:** Alessandra Borgognone, Maria Casadellà, María Martínez de Lagrán, Roger Paredes, Bonaventura Clotet, Mara Dierssen, Aleix Elizalde-Torrent

**Affiliations:** ^1^IrsiCaixa, Badalona, Spain; ^2^Center for Genomic Regulation, The Barcelona Institute for Science and Technology, Barcelona, Spain; ^3^Department of Infeccious Diseases and Immunity, University of Vic-Central University of Catalonia (UVic-UCC), Vic, Spain; ^4^CIBERINFEC, ISCIII, Madrid, Spain; ^5^Department of Pathology, Center for Global Health and Diseases, Case Western Reserve University, Cleveland, OH, United States; ^6^Department of Infectious Diseases Service, Germans Trias i Pujol University Hospital, Badalona, Spain; ^7^Fundació Lluita contra les Infeccions, Hospital Germans Trias i Pujol, Badalona, Spain; ^8^Department of Medicine and Life Sciences, Universitat Pompeu Fabra (UPF), Barcelona, Spain; ^9^Centro de Investigación Biomédica en Red de Enfermedades Raras (CIBERER), Barcelona, Spain

**Keywords:** LINE-1 retrotransposon, lamivudine, down syndrome, early senescence, cerebral cortex, hippocampus, transcriptomic profile

## Abstract

Elevated activity of retrotransposons is increasingly recognized to be implicated in a wide range of neurodegenerative and neurodevelopmental diseases, including Down syndrome (DS), which is the most common chromosomal condition causing intellectual disability globally. Previous research by our group has revealed that treatment with lamivudine, a reverse transcriptase inhibitor, improved neurobehavioral phenotypes and completely rescued hippocampal-dependent recognition memory in a DS mouse model, Ts65Dn. We hypothesized that retrotransposition rates would increase in the Ts65Dn mouse model, and lamivudine could block retrotransposons. We analyzed the differentially expressed long interspersed element-1 (LINE-1 or L1) mapping on MMU16 and 17, and showed for the first time that retrotransposition could be associated with Ts65Dn’s pathology, as misregulation of L1 was found in brain tissues associated with trisomy. In the cerebral cortex, 6 out of 26 upregulated L1s in trisomic treated mice were located in the telomeric region of MMU16 near *Ttc3*, *Kcnj6*, and *Dscam* genes. In the hippocampus, one upregulated L1 element in trisomic treated mice was located near the *Fgd4* gene on MMU16. Moreover, two downregulated L1s rescued after treatment with lamivudine were located in the intronic region of *Nrxn1* (MMU17) and *Snhg14* (MMU7), implicated in a variety of neurodegenerative disorders. To gain further insight into the mechanism of this improvement, we here analyzed the gene expression profile in the hippocampus and cerebral cortex of trisomic mice treated and no-treated with lamivudine compared to their wild-type littermates. We found that treatment with lamivudine rescued the expression of 24% of trisomic genes in the cortex (located on mouse chromosome (MMU) 16 and 17) and 15% in the hippocampus (located in the human chromosome 21 orthologous regions), with important DS candidate genes such as *App* and *Ets2*, rescued in both regions.

## 1 Introduction

Transposable elements (TEs) are mobile DNA sequences capable of integrating into the genome at a new site within the cell of its origin. Long interspersed element-1 (LINE-1, or L1) is the most successful transposable element family in human and constitutes ∼17% of the human genome (Lander et al., 2001). L1 elements are the only active autonomous retrotransposons in the human genome that encode the necessary “machinery” for their own mobilization. Studies have shown that during *in vitro* senescence, LINEs become de-repressed and start actively transposing, leading to higher levels of expression of L1 elements (De Cecco et al., 2019). Elevated retrotransposition rates have also been associated with several neurodegenerative conditions, including Alzheimer’s disease (AD), frontotemporal dementia, prion disease, and developmental disorders such as Rett’s syndrome, autism, or fragile X syndrome (Li et al., 2012), and has been suggested as a therapeutic target for premature aging. L1 copy numbers have been found to be dysregulated in patients with Rett syndrome (Muotri et al., 2010) and *Mecp2* knockout mice, a model of Rett syndrome (Lee et al., 2020). Moreover, L1 insertions are observed during neurogenesis, which is altered in Down syndrome (DS) mouse models (Pons-Espinal et al., 2013; Sierra et al., 2024) and mature neurons (Coufal et al., 2009; Macia et al., 2017). However, the comprehensive relationship between L1 and DS-associated brain alterations has not yet been investigated. DS is the most prevalent genetic disorder associated with moderate to severe intellectual disability due to a total or partial trisomy of the autosomal chromosome 21 (HAS21). DS is also recognized as a genetic form of AD (Dierssen, 2012) and is classified as a genetic progeroid syndrome in part due to the intriguing observation that the biological age of adults with DS consistently exceeds their chronological age by an average of 18.4 to 19.1 years throughout their lifespan (Murray et al., 2023). Notably, the early accumulation of L1 is a shared feature in both typical and atypical human progeroid syndromes (Della Valle et al., 2022).

Preclinical studies have shown that lamivudine [(−)-L-2′,3′-dideoxy-3′-thiacytidine (3TC)] improves cognition in senescence-accelerated prone 8 (SAMP8) mice (Li et al., 2021), and attenuates the progression of tauopathy upon the first symptoms of neuropathology in a mutant Tau transgenic mouse model (Vallés-Saiz et al., 2023). We postulate that the dysregulation of L1 elements contributes to genome instability, a hallmark of senescence and aging cells in DS, and may also affect gene expression. Treatment with lamivudine in the context of DS would help correcting these alterations. This hypothesis also stems from our previous research that has compellingly demonstrated that treatment with lamivudine mitigates DS-associated phenotypes in mice with the Ts65Dn mutation (Martinez de Lagran et al., 2022). Specifically, trisomy 21 mainly affects hippocampal-dependent cognitive function, recapitulated also in trisomic mice, and converging evidence suggests the hippocampus as a hub for dynamic TE expression and regulation. Importantly, lamivudine rescued both hippocampal and cerebral cortex-dependent phenotypes. Thus, our current work aims to analyze the gene expression profiles and L1 expression in the hippocampus and cerebral cortex of trisomic mice, both those that have received treatment with lamivudine and those that have not, as well as in wild-type littermates.

## 2 Methods

### 2.1 Animal models

Trisomic (Ts65Dn, TS) mice were obtained by crossing B6EiC4Sn.BLiA-Ts (1716) 65Dn/DnJ females with C57BL/6 × 6JolaHsd (B6C3F1/OlaHsd) hybrid males. The offspring was genotyped, and ∼25% had trisomy. Wild-type (WT) littermates were used as controls. After weaning, male mice were group-housed with three to four animals per cage in conventional 12:12 light cycle, controlled environmental conditions of humidity (50–70%) and temperature (21 ± 1°C), with water and standard rodent chow available *ad lib.* Experiments were conducted using 3–4 month-old male mice.

All experiments adhered to the principle of the “Three Rs”: replacement, reduction, and refinement, as outlined in Directive 63/2010 and its implementation in Member States. The study was conducted according to the guidelines of the local (law 32/2007) and European regulations (2010/63/EU) and the Standards for Use of Laboratory Animals no. A5388-01 (NIH) and was approved by the Ethics Committee of Parc de Recerca Biomèdica [Comité Ético de Experimentación Animal del PRBB (CEEA-PRBB); MDS18-0031]. Reporting followed the ARRIVE (Animal Research: Reporting of *In Vivo* Experiments) guidelines, with the modifications suggested by the Trisomy 21 Research Society for work with DS mouse models. The CRG is authorized to work with genetically modified organisms (A/ES/05/I-13 and A/ES/05/14). For all experiments, the experimenters were blinded to the genotype. The sample size was determined based on previous RNAseq studies that demonstrated sufficient statistical power.

### 2.2 Experimental design

**Figure 1:**
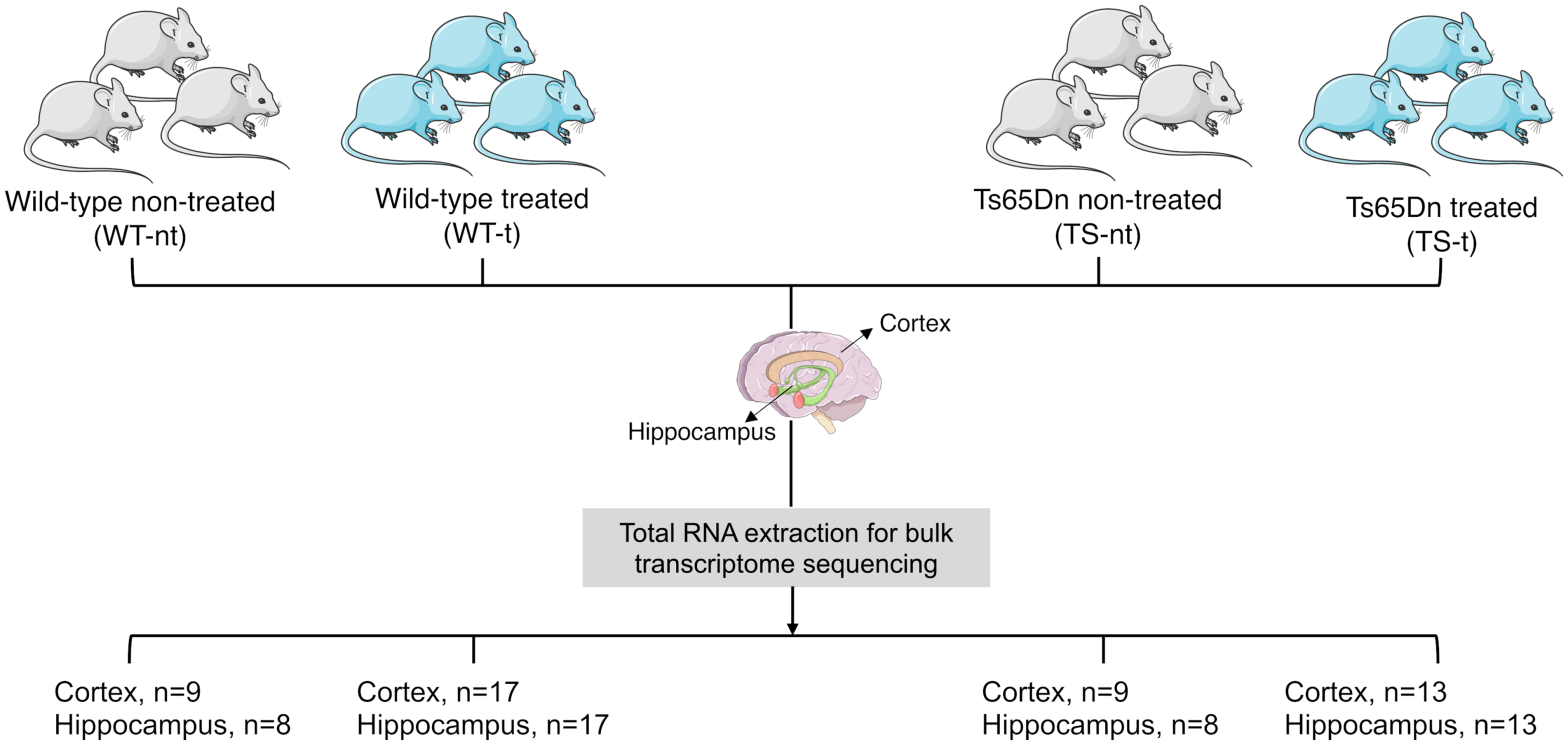
Study design and sample availability per mice group: treated (−t) or non-treated (−nt).

The tissues for the RNA seq experiments were obtained from a previous experiment already published (Martinez de Lagran et al., 2022). Briefly, TS and WT mice were assigned to either control conditions or to receive lamivudine 3 mg/kg (Epivir, 10 mg/mL, oral solution) in the drinking water, using simple randomization. 13 TS and 17 WT mice were treated with lamivudine, while 9 TS and 9 WT mice were used as the control group and received water. The treatment lasted for 4 months. We first analyzed differentially expressed long interspersed element-1 (LINE-1 or L1) mapping on MMU16 and 17, and possible misregulation of L1 in the studied tissues in the two different genotypes [trisomic (TS) or wild type (WT)]. We also conducted an analysis to determine the effect of lamivudine treatment on the mouse brain transcriptome. Our goal was to identify differentially expressed genes (DEGs) across different conditions (genotype and treatment). We obtained datasets from RNAseq experiments (total RNA for bulk transcriptome sequencing) from two different brain regions - hippocampus (HC) and cerebral cortex (CX) - for a total of 94 samples (sample size for each comparison group and tissue is shown in Figure 1).

### 2.3 RNA extraction

After the behavioral analysis, mice were sacrificed, and their cortices and hippocampi were dissected and frozen at −80°C. The samples were kept in dry ice or cold conditions as much as possible to preserve RNA integrity during the extraction process. To normalize the efficiency of tissue RNA extraction, frozen samples were weighed. Since the cortices were larger than the hippocampi, three parts of each cortex were used to ensure that all tissue fractions had approximately the same size and weight. Tissue was disrupted using TissueLyser, in a tube containing one 3 mm tungsten bead (Qiagen, Hilden, Germany) and 400ul of buffer RLT (Qiagen). The program used was 30 Hz for 30 s, 1 min stopped, and 25 Hz for 30 s more. Then, tubes were centrifuged at full speed for 3 min, followed by the RNeasy Plus Mini Kit protocol (Qiagen). Finally, we pooled samples in groups of three per condition and analyzed the RNA quality and concentration using the RNA 6000 Pico Kit (Agilent) in a Bioanalyzer equipment (Agilent). The RNA was frozen and kept at −80°C until it was sent to Macrogen (South Korea) for sequencing.

### 2.4 Bulk RNA sequencing and analysis of transcriptome data

Sequencing libraries were prepared by Macrogen using the Truseq Stranded Total RNA Library with Ribo-Zero Gold Kit (Illumina). This kit is designed to remove cytoplasmic and mitochondrial ribosomal RNA from samples while maintaining the strandedness of transcripts. The libraries were sequenced on a NovaSeq6000 platform (Illumina). The aim was to generate ∼ 100 M 150-BP paired-end reads per sample. After sequencing, Illumina adapters were removed and raw reads were trimmed for quality using *Trimmomatic v0.38* (Bolger et al., 2014) (ribosomal sequence were removed and % of bases with Phred score > 30 were discarded). A mean untrimmed read depth of ∼ 110 million reads/sample was yielded. Trimmed reads were then aligned to the mouse reference genome (GrCM38/mm10) using HISAT2 v2.1.0 (Kim et al., 2015) with recommended parameters, allowing up to 5 alignments per read (*−k 5*). For gene and transcript quantification, the aligned reads were assembled using StringTie v2.1.3b (Pertea et al., 2015). The locus-specific quantification of transposable elements (TE) expression was performed using Telescope (Bendall et al., 2019). Elements assigned to L1 sequences in the annotation were subsetted for further analysis. L1 elements of interest were visualized by the Integrative Genomics Viewer (IGV) (Thorvaldsdottir et al., 2013). The bioinformatic workflow for gene and TE transcriptome analysis is outlined in Supplementary Figure S1. All statistical tests were performed using the R software version 4.1.2 unless otherwise described.

### 2.5 Differential gene expression and functional enrichment analysis

Normalization and differential expression analysis between groups were performed using DESeq2 (Love et al., 2014), in R that utilizes a negative binomial model and Wald test. Genes and L1 elements having an adjusted *p*-value of ≤0.1 were considered differentially expressed, unless stated otherwise. Normalized counts extracted from DESeq2 were used for all statistical analyses. Gene Ontology (GO) and KEGG enrichment analysis (biological processes, cellular components, and molecular functions of potential targets) were performed with the clusterProfiler (Yu et al., 2012) R package. KEGG metabolic pathways were annotated based on the Kyoto Encyclopedia of Genes and Genomes (KEGG) databases^1^ and used for additional enrichment analyses. A raw *p*-value <0.05 and log2|fc| > =1.5 threshold was used to increase the number of elements (genes and LINE1s) in the differential expression analysis for the purpose of identifying potential candidate genes in a given comparison.

### 2.6 Protein–protein interaction (PPI) network construction

To explore the functional significance of the differentially expressed rescued genes (set of genes found differentially expressed exclusively in the comparison between TS-nt and WT-nt but not between TS-t and WT-nt mice after treatment with lamivudine), protein–protein interaction network was built using String 12.0.^2^ Active interaction sources, including experiments, databases, and co-expression as well as species limited to *Mus musculus*, an interaction score > 0.4, and hiding of disconnected nodes were applied in designed settings to construct the PPI networks.

### 2.7 Recognition memory measures

The mice used in the present study had undergone a Novel Object Recognition (NOR) test to evaluate the effects of lamivudine treatment on their recognition memory (Martinez de Lagran et al., 2022). To measure the mice’s performance in the test, a discrimination index (DI) was used. This index was calculated as [(time exploring the novel object – time exploring the familiar object)/total time of exploration] * 100 [see detailed methods in Martinez de Lagran et al. (2022)] at the end of a 4-month treatment with lamivudine (DI_4M) and as DI_4M – DI_baseline (delta DI). Higher DI values indicate better recognition memory performance (more time spent on the novel object). The results of this test were used to establish possible correlations with transcriptomic data.

Based on their performance in the NOR test, mice were classified as “good” [delta DI or DI_4M ≥ group mean + 3 standard error of mean (SEM)], “poor” (delta DI or DI_4M ≤ group mean – 3 SEM), and the remaining “intermediate” learners. Spearman’s correlation test was applied using the R ‘stat_cor’ and ‘lm’ functions to analyze associations between DI and rescued gene expression.

## 3 Results

After quality control and trimming, an average of 109,789,359 (±7,394,244) reads per sample were obtained. The principal component analysis of all comparison groups, based on gene expression (normalized counts) in both cortex and hippocampus, showed that samples did not clustered based on Treatment and Genotype (Supplementary Figure S2). All samples were kept in the analysis since the overall results were not affected by the removal of any outlier samples.

### 3.1 Trisomy leads to tissue-specific transcriptomic deregulation

**Figure 2:**
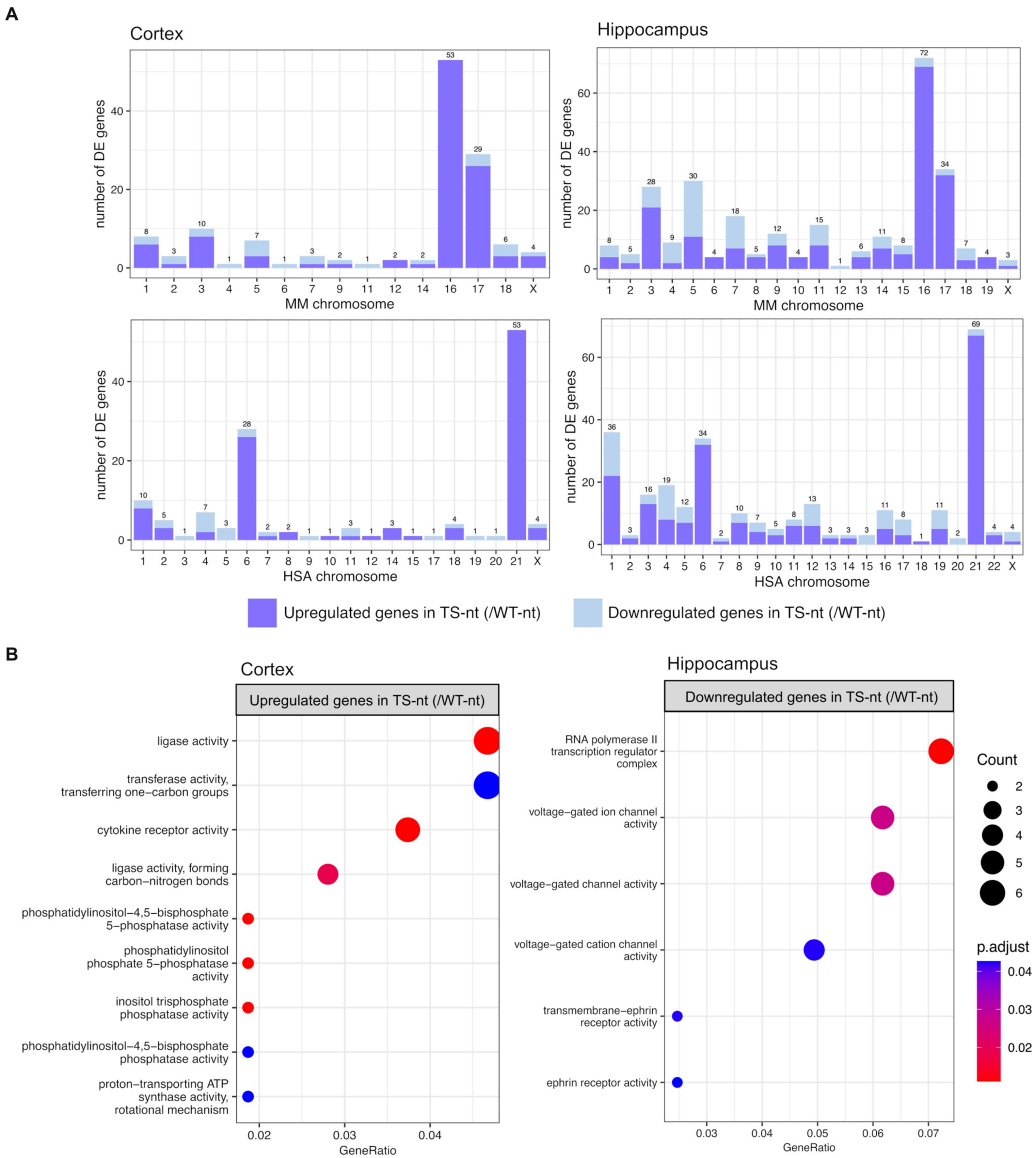
Chromosome mapping and gene ontology (GO) enrichment analysis for genotype effect (TS-nt vs. WT-nt). **(A)** Barplot for the number of differentially expressed genes per mouse chromosome mapping to their human orthologs identified in the cortex and hippocampus. **(B)** Enriched GO terms for upregulated genes in the cortex and downregulated genes in the hippocampus from the comparison between TS-nt vs. WT-nt mice. The dot size represents the number of genes. The color indicates the FDR value. A list of enriched GO terms and associated genes is provided in Supplementary Table S1.

In the comparison of genotype effects (TS-nt vs. WT-nt, adjusted *p*-value <0.1), we found 155 differentially expressed genes (DEGs) in the cortex (123 up- and 32 downregulated in TS-nt) and 315 DEGs in the hippocampus (226 up- and 89 downregulated in TS-nt) (Supplementary Table S1). Since DS is caused by the trisomy of HSA21, we sought to analyze the mouse chromosomal distribution of DEGs (TS-nt vs. WT-nt) that mapped to their human orthologs in both cortex and hippocampus (trisomic genes orthologous to HSA21 are located in mouse chromosome MMU 10, 16 and 17). We observed that the highest portion of DEGs, mostly upregulated, from comparison pairs TS-nt vs. WT-nt, were located on chromosomes MMU16 and 17 (Figure 2A and Supplementary Table S1). Also, we identified a genome-wide transcriptomic deregulation confirming our previous results (De Toma et al., 2021).

Gene enrichment analysis for upregulated genes in the cortex of TS-nt (vs. WT-nt) mice displayed functions mostly related to ligase activity, phosphatidylinositol, inositol phosphatase, and cytokine receptor activities (Figure 2B and Supplementary Figure S3 and Supplementary Table S1). Downregulated genes in the hippocampus of TS-nt (vs. WT-nt) mice were enriched in functions related to ion channel activity and ephrin receptor (Figure 2B and Supplementary Figure S3 and Supplementary Table S1). However, no enriched GO functions were identified for downregulated genes in the cortex nor for upregulated genes in the hippocampus in the comparison between TS-nt and WT-nt mice.

### 3.2 Transcriptional profiling of LINE-1 retrotransposons

To gain an overall understanding of potential changes in the transcriptional activity of L1s, we quantified and compared the expression of L1 elements in the different experimental conditions using the bioinformatics tool Telescope (see Methods). The Principal Component Analysis performed on normalized L1 counts did not show a clear clustering (Supplementary Figure S4A), similarly to what observed in gene expression (Supplementary Figure S2). We then compared the proportion of L1 transcripts by counting RNA-seq reads mapped on L1 elements over total transcriptome reads for each sample. We found no significant difference in the proportion of L1 transcript over total RNA counts between groups in any of the two tissue types (Supplementary Figure S4B).

**Figure 3:**
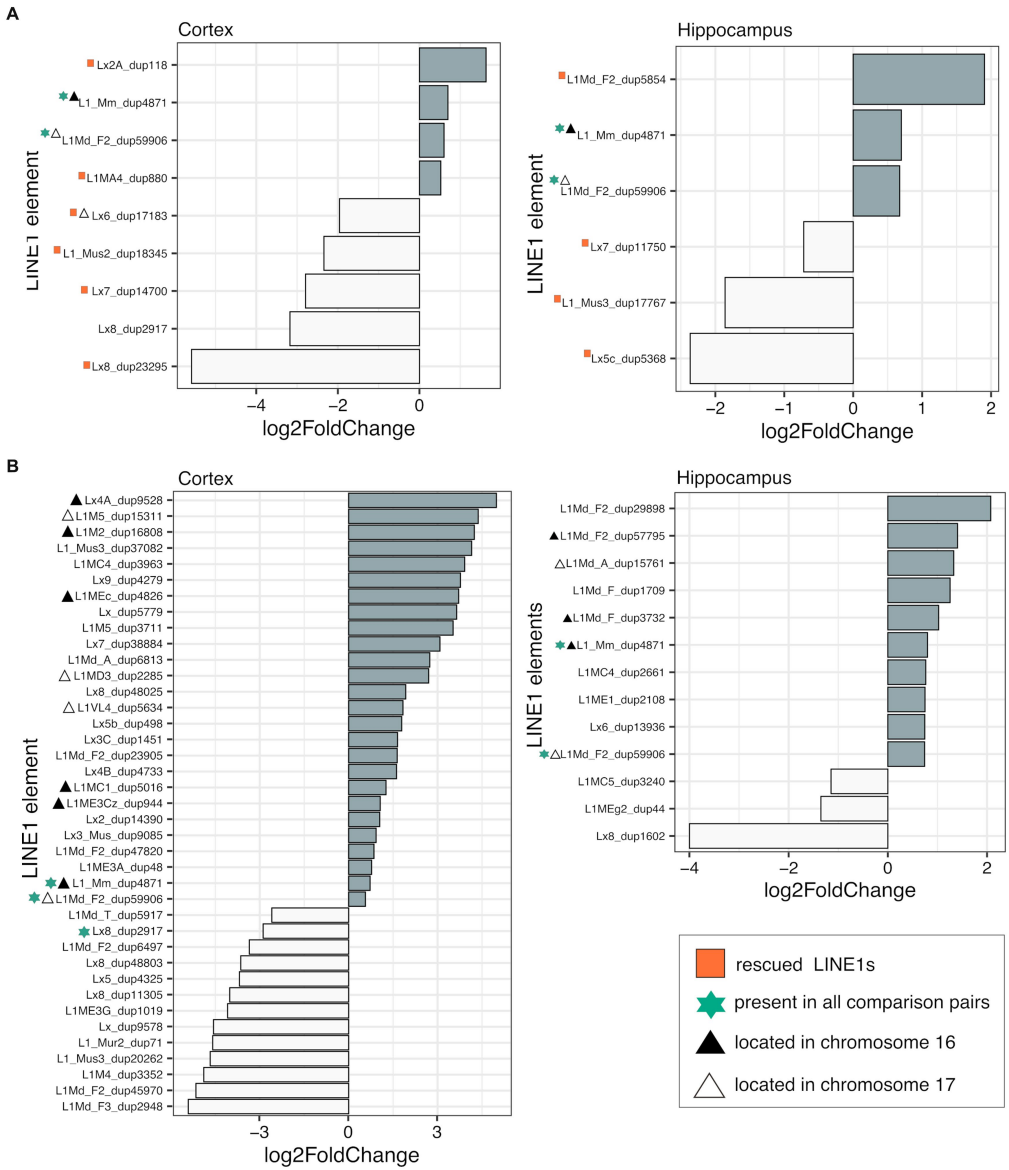
Differentially expressed LINE-1 s. Differential expression (adjusted *p*-value <0.1) of LINE-1 elements assessed in **(A)** genotype effect (TS-nt vs. WT-nt) and **(B)** DE LINE-1 s in the TS-t vs. WT-nt comparison. Rescued LINE-1 elements are marked with red squares. Element size and chromosome location are provided in Supplementary Table S2.

To assess the impact of genotype on L1 transcription in the cortex and hippocampus, we conducted differential expression analyses between TS-nt vs. WT-nt mice. We found significant changes in expression (adjusted *p*-value <0.1) in 9 (4 up- and 5 downregulated in TS-nt) and 6 (3 up- and 3 downregulated in TS-nt) L1s in the cortex and hippocampus, respectively (Figure 3A). Additionally, we observed 3 differentially expressed (DE) L1s located on the mouse chromosomes MMU16 and 17 in both cortex and hippocampus (Supplementary Figure S5 and Supplementary Table S2).

In contrast, evaluation of the treatment effect in the TS and WT comparison pairs for each tissue showed only one DE L1 in the hippocampus of WT mice (WT-t vs. WT-nt), located on chromosome MMU1 (Supplementary Table S2).

In the comparison between TS-t vs. WT-nt mice we found 39 (26 up- and 13 downregulated in TS-t) and 13 (10 up- and 3 downregulated in TS-t) DE L1s in the cortex and hippocampus, respectively (Figure 3B). Of these, 10/26 (cortex) and 6/10 (hippocampus) upregulated L1s mapped in the mouse chromosomes MMU16 and 17 (Supplementary Figure S6 and Supplementary Table S2), with a subgroup of elements located in the telomeric region of MMU16 and close to genes *Ttc3*, *Kcnj6* and *Dscam* (Supplementary Figure S6).

Therefore, we evaluated the rescuing effects of lamivudine on the cerebral cortex and hippocampus of trisomic mice by analyzing whether L1s deregulated in TS-nt were restored to WT levels in TS-t mice. We identified 6 and 4 rescued L1s after treatment with lamivudine in the cortex and hippocampus, respectively (Supplementary Figure S7 and Supplementary Table S2), located in different regions across the mouse chromosomes and in close proximity to genes *Emc7, Scm3, Ints14, Cdh12, Nrxn1* (cortex) and *Snhg14* (hippocampus) (Supplementary Figure S8). We also found that a number of misregulated L1s contained LINE1-encoded reverse transcriptase to promote transposition in the brain cells, across the comparison groups presented above (Supplementary Table S2).

Finally, we assessed potential associations between misregulated LINE1s (Figure 3) and senescence markers present in the gene set from our Ts65Dn mouse model (Supplementary Table S3). In the correlation analysis between rescued LINE1s (Supplementary Figure S9), differentially expressed LINE1s in TS-t/WT-nt (Supplementary Figure S10) and marker expression levels, we did not observe similar pattern between tissues. However, strong associations between LINE1s and senescence-associated markers, including p53 (*Trp53*), β-galactosidase (*Glb1*), secretory phenotype (*Cxcl12*) and calcium binding protein A4 (*S100a4*), were detected in all correlation analyses.

### 3.3 Treatment effect

When analyzing the effect of lamivudine treatment on gene expression profiles in the hippocampus and the cortex of Ts65Dn and WT mice, we did not observe any distinct PCA clusters affected by the treatment, suggesting only modest changes in the global mRNA expression (Supplementary Figure S11). After filtering for an adjusted *p*-value <0.1, we obtained a small number of DE genes, which were mostly classified as pseudogenes and lncRNAs (Supplementary Table S4). By adjusting to a raw *p*-value <0.05 and log2|fc| > =1.5, we found 131 and 187 DEGs between TS-t and TS-nt mice in the cortex and hippocampus, respectively. In the same way, the treatment induced changes in WT, with 190 and 169 DEGs when comparing WT-t vs. WT-nt mice in the cortex and hippocampus, respectively (Supplementary Table S4). Again, most DEGs genes were classified as lncRNA and pseudogenes (Kapusta et al., 2013) ~ 35% of the treatment-related DEGs were HSA21 orthologs in both WT and trisomic mice (Supplementary Table S4), having variable distribution across the mouse chromosomes (Supplementary Figure S12).

**Figure 4:**
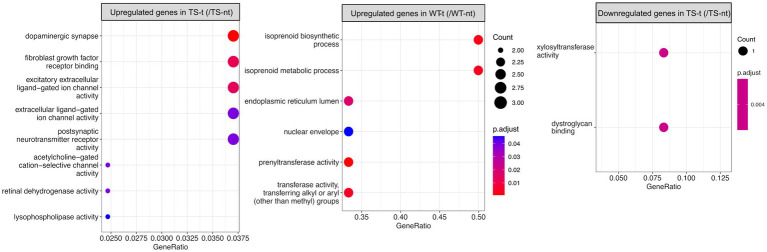
Gene ontology (GO) enrichment analysis for differentially expressed genes in treatment effect comparison pairs (hippocampus). Enriched GO terms for treatment effect comparison pairs (TS-t vs. TS-nt and WT-t vs. WT-nt) are shown (raw *p*-value <0.05 and log2|fc| > =1.5). The x-axis represents the percentage of genes in a given GO term expressed as ‘gene ratio’. The size of the dots represents the number of genes. The color indicates the FDR value. A list of enriched GO terms and associated genes is provided in Supplementary Table S4.

In the hippocampus, upregulated genes of Ts65Dn mice treated with lamivudine (vs. TS-nt) were enriched in functions related to synaptic and ion channel activities, while these GO terms were not found in WT-treated mice (vs. WT-nt) (Figure 4 and Supplementary Table S4). In the cortex, no functional enrichment was found for DEGs associated with lamivudine treatment in TS or WT.

### 3.4 Rescuing effect of lamivudine

**Figure 5:**
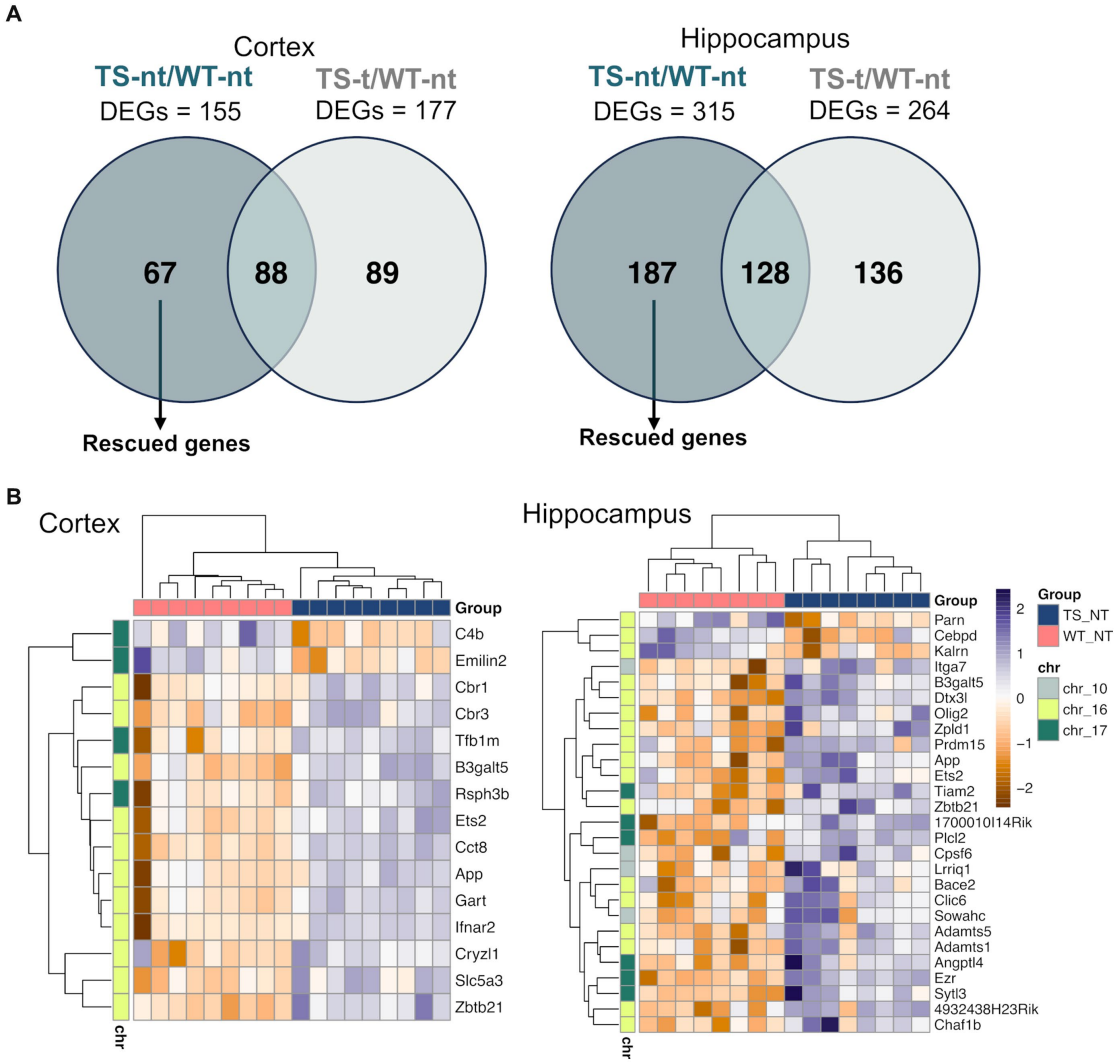
Expression of rescued genes after lamivudine treatment in the cortex and hippocampus. **(A)** Venn diagrams showing genes that were restored (rescued) after treatment with lamivudine (those altered by genotype that are not present in treated mice and correct their levels to WTs). **(B)** Heatmap representation of rescued gene expression (adjusted *p*-value <0.1) in cortex and hippocampus. Gene and sample-wise hierarchical clustering was performed on individual normalized read counts (rlog). The input matrix was scaled in rows to visualize changes in expression on the gene level and columns to display the relatedness of samples. A list of gene names for rescued genes is provided in Supplementary Table S5.

In our study, we also analyzed genes that were rescued by lamivudine treatment, which were defined as those differentially expressed in TS-nt vs. WT-nt but not in TS-t vs. WT-nt (Figure 5A). We identified 67 and 187 rescued genes in the cortex and hippocampus (Figure 5A and Supplementary Table S5), of which, 24 and 15% respectively, mapped to the mouse orthologue regions (MMU10, MMU16, and MMU17) of the HSA21 (Supplementary Figure S13 and Table S5). Among these, rescued genes were mostly associated with neuronal alterations (Figure 5B and Supplementary Figure S14), such as *App*, *Bace2* and *Olig2* (Supplementary Table S5), with 4 genes common to both tissues (*Zbtb21:* zinc finger and BTB domain containing 21, *App*: amyloid beta (A4) precursor protein, *Ets2*: E26 avian leukemia oncogene 2, 3′ domain and *B3galt5*: UDP-Gal:betaGlcNAc beta 1,3-galactosyltransferase, polypeptide 5).

**Figure 6:**
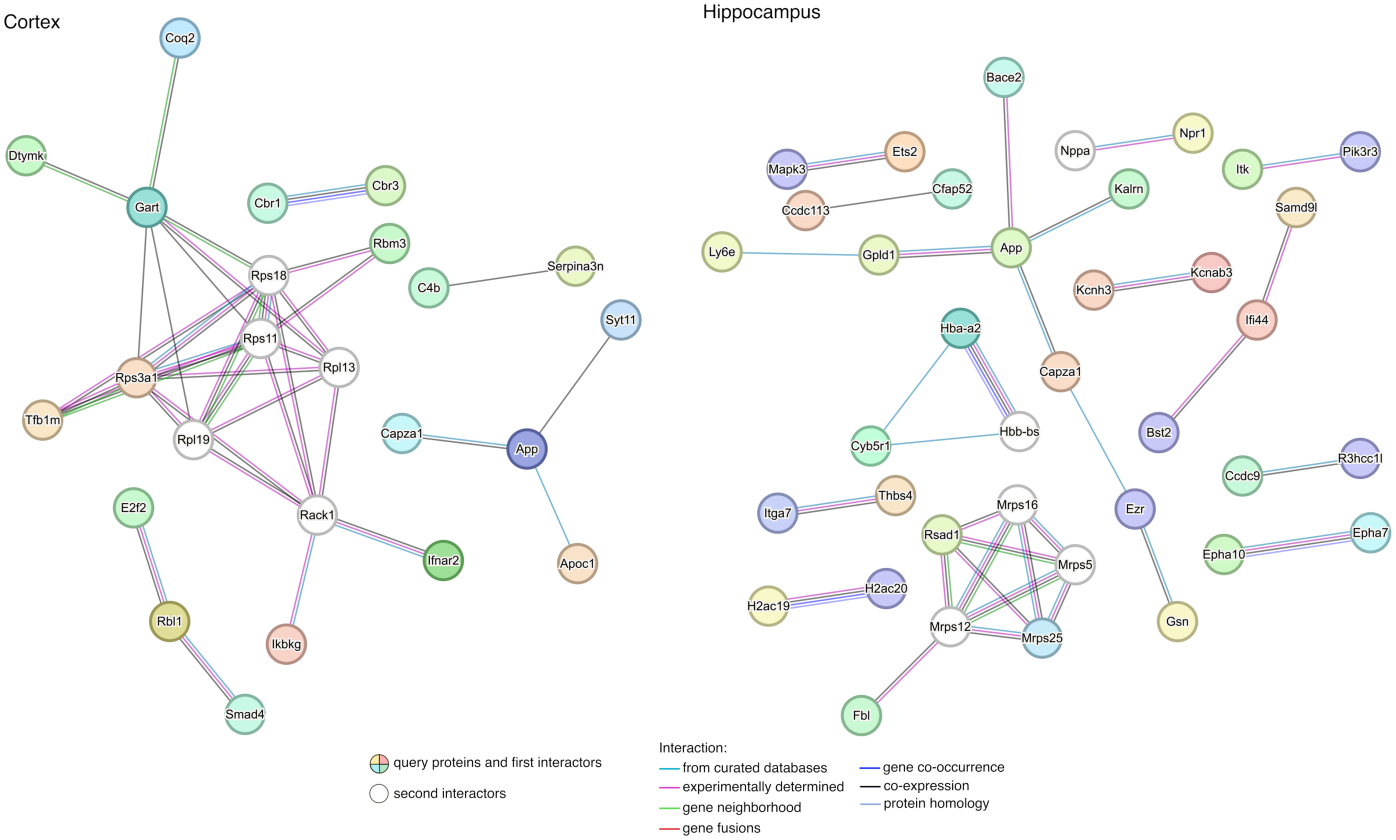
Protein–protein interaction (PPI) network for rescued genes in the cortex and the hippocampus. The lists of rescued genes were submitted to STRING (see footnote 2) for searching PPIs. Disconnected nodes in the network were excluded from the plot. Nodes represent genes while edges represent PPIs between two genes. Details of enriched functions are provided in Supplementary Table S6.

To gain a better understanding of the interactions among rescued genes, we constructed protein–protein interaction (PPI) networks with the 67 and 187 genes, including first- and second-degree interactors (STRING database). After removing non-interacting proteins, 5 and 13 clusters of highly interconnected nodes (proteins) were identified in the cortex and hippocampus (Figure 6), respectively, suggesting that the implicated genes were functionally related (Supplementary Table S6).

When exploring the biological meaning of the main subnets of the networks, we found enrichment in functions related to DS in the cortex, such as regulation of neuron death, neuron apoptotic process, learning, memory, and cognition, among other general processes (Supplementary Table S6). In contrast, no significant functional enrichment was detected in genes contributing to the PPI from rescued genes characterized in the hippocampus.

### 3.5 Additional possible lamivudine mechanisms

A molecular analysis comparing gene expression patterns in treated trisomic brains and wild-type brains can provide information on how the treatment influences gene regulation. This can help identify specific genes that may be involved in the pathophysiology of trisomy and gain more insight into possible mechanisms underlying lamivudine action. We identified a total of 177 DEGs in the cortex (126 up- and 51 downregulated in TS-t) and 264 DEGs in the hippocampus (204 up- and 60 downregulated in TS-t) (Supplementary Table S7). We observed that most of the DEGs in both tissues (73 in the cortex and 92 in the hippocampus) were located on the mouse chromosomes MMU 16 and 17 (Supplementary Figure S15 and Supplementary Table S7).

**Figure 7:**
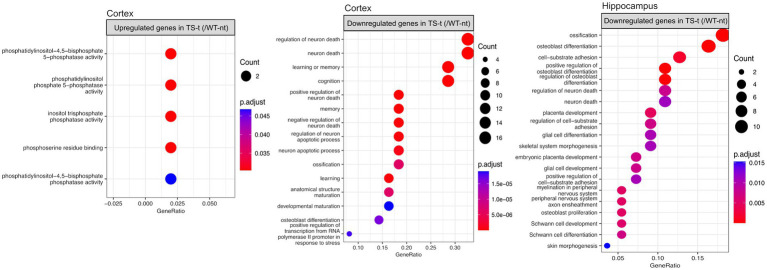
Gene ontology (GO) enrichment analysis for differentially expressed genes in the comparison between TS-t vs. WT-nt mice. Enriched GO terms for cognitively rescued genes in the cortex and hippocampus (raw *p*-value <0.05 and log2|fc| > =1.5). The x-axis represents the percentage of genes in a given GO term expressed as ‘gene ratio’. The size of the dots represents the number of genes. The color indicates the FDR value. A list of enriched GO terms and associated genes is provided in Supplementary Table S7.

When exploring the functional enrichment for the set of DEGs, we found that downregulated genes in TS-t vs. WT-nt mice were mainly enriched in GO functions related to cognition and regulation of neuron death, in both cortex and hippocampus (Figure 7, Supplementary Figure S16 and Supplementary Table S7). Moreover, enriched KEGG pathways in cortex were found related to Alzheimer’s disease, TNF and IL-17 signaling pathways, among others (Supplementary Figure S16 and Supplementary Table S7).

Also, similar to the enriched GO terms found in the comparison between TS-nt vs. WT-nt mice (Figure 2B), upregulated genes in the cortex of TS-t vs. WT-nt mice displayed GO functions mostly related to phosphatidylinositol and inositol phosphatase activities (Figure 7, and Supplementary Table S7).

### 3.6 Expression of rescued genes by lamivudine treatment correlated with cognitive improvement

**Figure 8:**
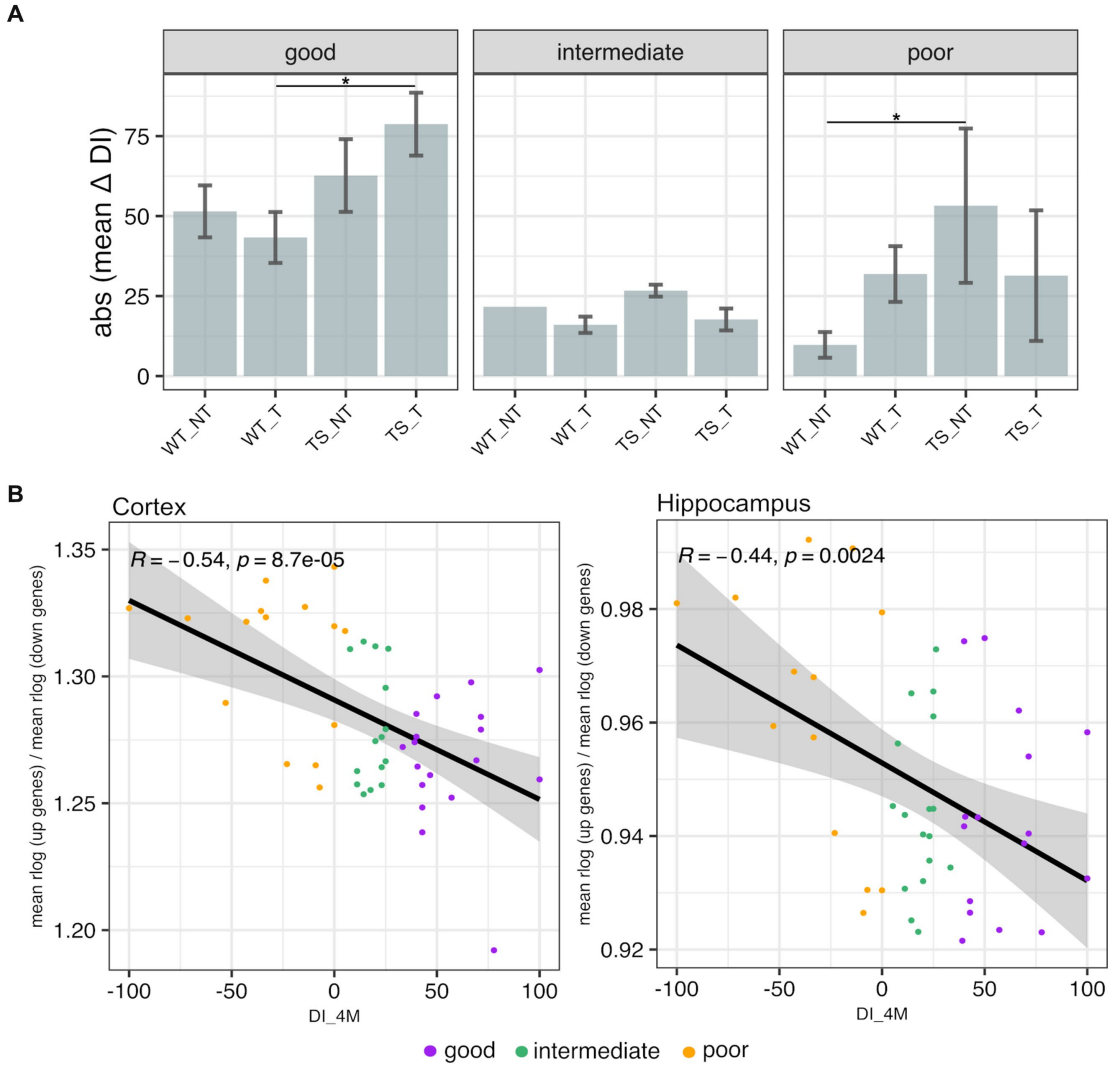
Novel object recognition memory and transcriptional profiling. **(A)** Absolute mean values of delta discrimination index (DI_4M – DI_baseline) ± 3 standard errors of the mean (SEM) in TS and WT mice. Error bars represent SEM. Asterisks denote the level of significance (^*^p < 0.05). **(B)** Scatterplots showing Spearman’s correlations between discrimination index after 4 months of treatment (DI_4M) and rescued gene ratio (mean of normalized upregulated rescued gene expression / mean of normalized downregulated rescued gene expression, across all mouse chromosomes) evaluated in cortex and hippocampus of mice.

To classify TS and WT mice based on recognition memory impairment, we used a discrimination index (DI) − ranging from −100 (only exploration of the familiar object) to 100 (only exploration of the novel object) – following criteria as detailed in Methods section. Our results showed that TS mice treated with lamivudine (TS-t) had higher delta DI values (mean = 78.6) and were mostly categorized as ‘good’, with statistically significant differences in delta DI compared to WT-t mice in the ‘good’ category (*p* = 0.021, Wilcoxon test) (Figure 8A). In contrast, TS-nt mice in the ‘poor’ category exhibited the lowest delta DI values (mean = −53.3), showing significant differences with the WT-nt group (*p* = 0.030, Wilcoxon test) (Figure 8A). We next sought to explore possible correlations between the transcriptional levels of rescued genes identified in this study (Supplementary Table S5) and the DI evaluated after 4 months of treatment (DI_4M), representing the best learning point. Our findings revealed that the expression of upregulated rescued genes showed a negative correlation with DI_4M in both cortex (Supplementary Figure S17) and hippocampus (Supplementary Figure S18), while those rescued genes that were downregulated showed an opposite trend. A similar trend was observed after calculating a gene expression index (as detailed in Figure 8B legend), where the expression of genes rescued by treatment with lamivudine, associated to neurocognitive disorders (Supplementary Table S5), negatively correlated with the DI_4M (Figure 8B). Moreover, mice classified as ‘poor’ tended to show a negative correlation between rescued gene expression ratio and DI_4M, with marginal statistical significance in hippocampus (Supplementary Figure S19).

## 4 Discussion

In this study, we conducted a comprehensive transcriptomic analysis of the cerebral cortex and hippocampus in a Down syndrome mouse model, Ts65Dn, to investigate the potential impact of lamivudine on genes associated with neural function.

We identified genome-wide transcriptomic deregulation in TS-nt compared to WT-nt, confirming our previous results (De Toma et al., 2021). Of note, the highest portion of DEGs, mostly upregulated, mapped to chromosomes MMU 10, 16, and 17, orthologous to HAS21, in both cortex and hippocampus. Gene enrichment analysis for upregulated genes in the cortex of TS-nt (vs. WT-nt) mice displayed functions mostly related to ligase activity, phosphatidylinositol, inositol phosphatase, and cytokine receptor activities. On the other hand, downregulated genes in the hippocampus of TS-nt mice were enriched in functions related to ion channel activity and ephrin receptor. Our findings suggest that only modest changes in global mRNA expression may be attributed to treatment with lamivudine. However, in the hippocampus of Ts65Dn mice treated with lamivudine (TS-t vs. TS-nt), upregulated genes were notably enriched in functions related to synaptic, neurotransmitter receptor and ion channel activities, while these GO terms were not found in WT-treated mice (WT-t vs. WT-nt).

Given that nucleoside reverse transcriptase inhibitors (NRTIs) have been used to suppress retrotransposition (Banuelos-Sanchez et al., 2019), and the fact that transposable elements have recently gained attention as potential contributors to neurocognitive disorders, we also quantified and compared the expression of L1 elements in the different experimental conditions. It has been reported that when L1 is transcriptionally derepressed, it triggers an IFN-I response, which is a phenotype of late senescence (Antón-Fernández et al., 2023; Vallés-Saiz et al., 2023). Of note, uncontrolled activation of the IFN pathway is present in DS, due to overexpression of three IFN genes, and can cause neuroinflammation and neurotoxicity, which are primary hallmarks of DS (Chung et al., 2021).

As part of a new approach to address neurocognitive impairment in neurodegenerative diseases, lamivudine has been repurposed to target the increased activity of retrotransposable elements observed in cell senescence and aging. L1s can contribute to genetic instability by generating target site deletions, insertions of flanking DNA, recombination with other retrotransposons, and the possible generation of chromosomal inversions and interchromosomal translocations (Kazazian, 2004; Goodier and Kazazian, 2008; Beck et al., 2011; Hancks and Kazazian, 2012). In addition, inactive L1 elements (unable to mobilize) can contain mutations in their sequence and express truncated proteins, causing varying levels of cell toxicity and DNA damage (Kines et al., 2014). Moreover, L1 elements can alter the dynamic of the neuronal transcriptome and modulate the expression pattern of several nearby genes (Chung et al., 2021), by inducing alternative splicing, adenylation signals and transcription factor-binding sites. In fact, growing evidence suggests that misregulation of L1s in brain tissues is associated with neurological disorders (Terry and Devine, 2020).

When assessing the impact of genotype on L1 transcription in the cortex and hippocampus, we found significant changes in the expression of 9 (4 up- and 5 downregulated in TS-nt) and 6 (3 up- and 3 downregulated in TS-nt) L1s in the cortex and hippocampus, respectively, with a group of DE L1s located on the mouse chromosomes MMU16 and 17. Our results might provide indirect evidence for misregulation of L1 retrotransposons in the Ts65Dn mouse model, potentially contributing to some pathological aspects. Considering the altered adaptive and regenerative capacities in the brain of individuals with DS, the implications of these somatic insertions could be of profound significance.

Interestingly, in this study, L1 insertions on MMU16 were located near the DEGs of the cerebral cortex of TS mice. Specifically, we found L1 insertions in the distal MMU16 that includes a region of conserved linkage with HSA21 (Cabin et al., 1998). This region extends from 21q11.1 to 21q22.3 and is limited by *STCH*, the proximal-most known gene on HSA 21, and *Mx*, the distal-most known gene on MMU16. Twenty-four genes define this conserved linkage with no discordances in the order or presence of genes in this region. Some nearby genes include Dscam, Kcnj6, and Ttc3. *Dscam* (Down syndrome cell adhesion molecule) (Yamakawa, 1998), located in humans in 21q22.2–22.3, is critical for many of the neurological phenotypes of DS. For example, DSCAM protein is a new class of neural cell adhesion molecules involved in the development of the nervous system and with an important role in neural wiring and synaptogenesis (Sterne et al., 2015). *Kcnj6* encodes for a potassium inwardly rectifying channel subfamily J member 6 and contributes to synaptic and cognitive abnormalities in DS (Kleschevnikov et al., 2017). Finally, *Ttc3* (tetrapeptide repeat domain 3) is located within the DS critical region, and overexpression of TTC3 can accelerate cognitive decline, but the specific mechanism is unknown (Zhou et al., 2022). One L1 element upregulated in the hippocampus of TS-t (vs. WT-nt) mice was located near the *Fgd4* gene on MMU16. *Fgd4* has been identified as a susceptibility gene for amyotrophic lateral sclerosis (Wei et al., 2019), and also associated with Charcot–Marie–Tooth disease (Zis et al., 2017).

Regarding rescued L1s after treatment with lamivudine, we found two elements downregulated and in the proximity of the intronic region of *Nrxn1* (MMU17) and *Snh14* (MMU7) genes. Interestingly, deletion of *Nrxn1* (neurexin1), having a role in formation of functional synaptic structures, has been related to various neuropsychiatric disorders such as autism spectrum disorder, intellectual disability, and schizophrenia (Cuttler et al., 2021).

Also, emerging evidence suggest that *Snh14* (lncRNA-small nucleolar RNA host gene 14), a gene for long non-coding RNAs, is involved in neurological impairment and inflammatory response in various neurological disorders (Zhong et al., 2019). The constantly evolving landscape of research underscores the need for continued exploration of L1 dynamics to unravel the complexities of neurodevelopmental and neurodegenerative disorders.

We also analyzed the rescued genes, defined as those differentially expressed in TS-nt vs. WT-nt but not in TS-t vs. WT-nt. A group of genes rescued by treatment with lamivudine located on the mouse chromosomes orthologous to HSA21, MMUs 10, 16 and 17. Interestingly, rescued genes upregulated in both cortex and hippocampus included genes related to DS and Alzheimer disease, such as *App* (Tosh et al., 2021), *Ets2* (Helguera et al., 2005), *Bace2* (Alić et al., 2021), and *Olig2* (Choong et al., 2015). *App*, which encodes amyloid precursor protein (APP), explains the dramatically increased susceptibility to early-onset AD in individuals with DS. By the age of 40 years and beyond, virtually all individuals with DS show AD-associated neuropathology including brain deposition of amyloid-β (Aβ) protein in neuritic plaques and neurofibrillary tangles. *Ets2* upregulation in susceptible neurons promotes the activation of a mitochondrial-dependent proapoptotic pathway of cell death in DS and AD (Helguera et al., 2005). *Bace2* encodes an integral membrane glycoprotein that plays a crucial role in the cleavage of amyloid precursor protein into amyloid beta peptide – an essential step in the etiology of AD and DS (Gomez et al., 2020). *Olig2* encodes a protein that is essential for oligodendrocyte and motor neuron specification. Olig2 triplication causes developmental brain defects in DS (Chakrabarti et al., 2010; Lu et al., 2012; Xu et al., 2019). Olig2 collaborates with ZNF488 to promote oligodendrocyte differentiation (Zong et al., 2019). Upon further examination of the rescued genes located in MMU 16 and 17 in the cortex, additional candidates were found such as *Cct8* (Chaperonin containing TCP1 subunit 8), *Rsph3b* (Radial spoke 3B homolog), and *C4b* (Complement C4B).

The main subnets of the PPI networks showed enrichment in functions related to DS in the cortex. *App* expression is enriched in MMU16 chromosome and APP protein is interacting with SYT11 and CAPZA1. SYT11 (Synaptotagmin 11) plays an important role in dopamine transmission by regulating endocytosis and the vesicle-recycling process and is crucial for neurodevelopment and synaptic plasticity. Moreover, it inhibits the conventional cytokine secretion of at least IL-6 and TNF and phagocytosis in macrophages and microglia. CAPZA1 (Capping actin protein of muscle Z-line subunit alpha 1), which also interacts with APP, may play a role in the epithelial cell junctions formation. We have also observed that, in the hippocampus, APP and BACE2 are interacting, along with CAPZA1 and GPLD1 (Glycosylphosphatidylinositol specific phospholipase D1), among others.

During the comparison of the groups TS-t vs. WT-nt, it was found that the downregulated genes in both cortex and hippocampus were enriched in GO terms related to various neuronal functions - such as regulation of neuron death, glial cell development, memory, cognition and Schwann cell development - suggesting that treatment with lamivudine in TS mice may modulate the expression of genes involved in a wide range of neuronal activities.

Furthermore, enriched KEGG pathways in cortex were found to be related to: (i) Alzheimer’s disease; (ii) Il-17 signaling pathway: several studies have suggested a critical role for the interleukin-17A (IL-17A) cytokine family in human inflammatory or autoimmune diseases and neurodegenerative diseases (Chen et al., 2020). Although the specific mechanism of IL-17A in neurodegenerative diseases is still controversial, it is generally accepted that IL-17A causes diseases by activating glial cells; (iii) TNF signaling pathway: the TNF signaling pathway plays an essential role in various physiological and pathological processes, including cell proliferation, differentiation, apoptosis, and modulation of immune responses and induction of inflammation. These results suggest that genes involved in Alzheimer’s neuropathology and neuroinflammation pathways were downregulated in Ts65Dn mice treated with lamivudine.

Of note, *Ddit4*, downregulated in TS-t vs. WT-nt and located in chromosome 10, is the coding gene for RTP801/REDD1. RTP801, initially recognized for its involvement in cellular stress responses and regulation of mTOR signaling, has gained attention in the context of neurodegenerative diseases (Pérez-Sisqués et al., 2021). Studies suggest that RTP801/REDD1 may play a role in modulating pathways associated with neuroinflammation, apoptosis, and synaptic function in the context of Alzheimer’s disease (AD). Dysregulation of these processes is implicated in the neuronal loss and cognitive decline observed in AD. RTP801/REDD1 might influence mTOR signaling, which is a pathway crucial for cellular homeostasis. Alterations in mTOR signaling have been reported in AD (Malagelada et al., 2011).

In our current research, we utilized behavioral testing from a previous study (Martinez de Lagran et al., 2022) to assess the impact of lamivudine treatment on recognition memory. When the performance of mice classified as ‘good’, ‘intermediate’ and ‘poor’ was compared between the experimental groups, the highest scores were observed in ‘good’ TS-t mice. In contrast, TS-nt ‘poor’ mice exhibited the weakest performance. In addition to genotype and treatment, reasons for the individual and group-dependent variation in recognition memory may be related to mice with poor recognition memory at baseline taking significantly longer than other animals to achieve the final behavioral output. Correlation analyses of behavioral testing score and expression of genes rescued by treatment with lamivudine in both cortex and hippocampus suggested that mice with poor recognition memory had higher expression of rescued genes, such as *App, Ets2 and CCt8* (Sultan et al., 2007), which have previously been linked to DS alterations. Although further investigation is needed to understand the mechanism behind these associations, these results provide a potential regulation landscape linking the transcriptional potential to behavior in the Ts65Dn mouse model.

Our work conducted on the Ts65Dn mouse model shows for the first time that retrotransposition could be associated with DS, as misregulation of L1 was found in brain tissues associated with trisomy. Moreover it has uncovered novel transcriptional signatures in the hippocampus and cerebral cortex. Our findings propose a link between L1 misregulation and changes in gene expression profiles. Although, we fully acknowledge the intrinsic limitation of the design presented in this study which did not allow experimental validation of sequencing results.

Insights into the dysregulation of L1 in affected brain regions highlight the potential role of these retrotransposons in disease progression. The knowledge gained from these findings holds the potential to suggest therapeutic strategies and improve current understanding of the molecular underpinnings. Promising therapeutic interventions like reverse transcriptase inhibitors are being explored to improve neurobehavioral phenotypes and reverse cognitive deficits (Sullivan et al., 2024).

## Data availability statement

The raw RNA-seq data and datasets presented in this study can be found at GEO (GEO accession number: GSE252135). The original code has been deposited in Github (https://github.com/MicrobialGenomics-IrsicaixaOrg/Lamivudine_mice_RNAseq.git).

## Ethics statement

The animal study was approved by Ethics Committee of Parc de Recerca Biomèdica (Comité Ético de Experimentación Animal del PRBB (CEEA-PRBB); MDS 18-0031). The study was conducted in accordance with the local legislation and institutional requirements.

## Author contributions

AB: Writing – original draft, Software, Methodology, Formal analysis, Conceptualization. MC: Writing – original draft, Methodology, Investigation. MM: Writing – review & editing, Data curation. RP: Writing – review & editing, Supervision, Resources, Funding acquisition. BC: Writing – review & editing, Supervision, Conceptualization. MD: Writing – original draft, Supervision, Methodology, Conceptualization. AE-T: Writing – original draft, Methodology, Investigation, Conceptualization.

## Footnotes

^1^ http://www.genome.jp/kegg/

^2^ http://string-db.org/
