# Supplementary material for: Lamivudine modulates the expression of neurological impairment-related genes and LINE-1 retrotransposons in brain tissues of a Down syndrome mouse model
Source: Front Aging Neurosci. 2024 Jul 19;16:1386944. doi: 10.3389/fnagi.2024.1386944 (PMC11294114; doi:10.3389/fnagi.2024.1386944)
Supplement: Supplementary file 8 [file Data_Sheet_1.docx]

Supplementary Material

**Figure S1.** Bioinformatics workflow for genes and LINE-1 element expression in mice

**
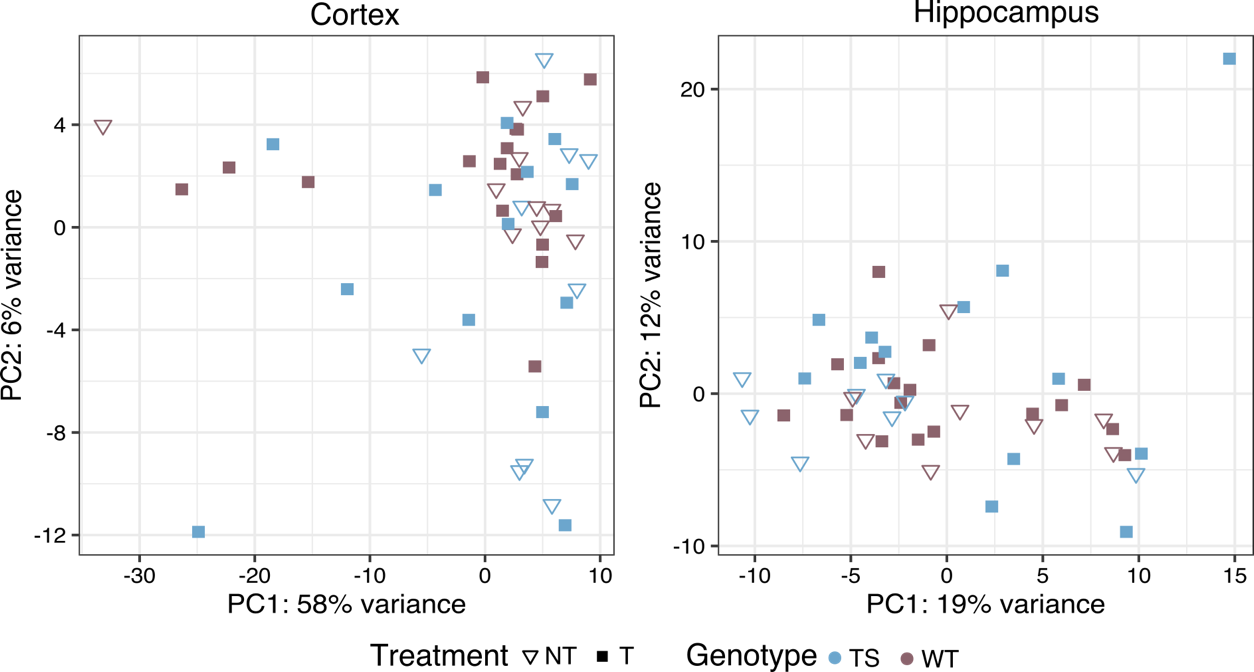
**

**Figure S2.** Principal component analysis based on gene expression by RNA sequencing in cortex and hippocampus.

**
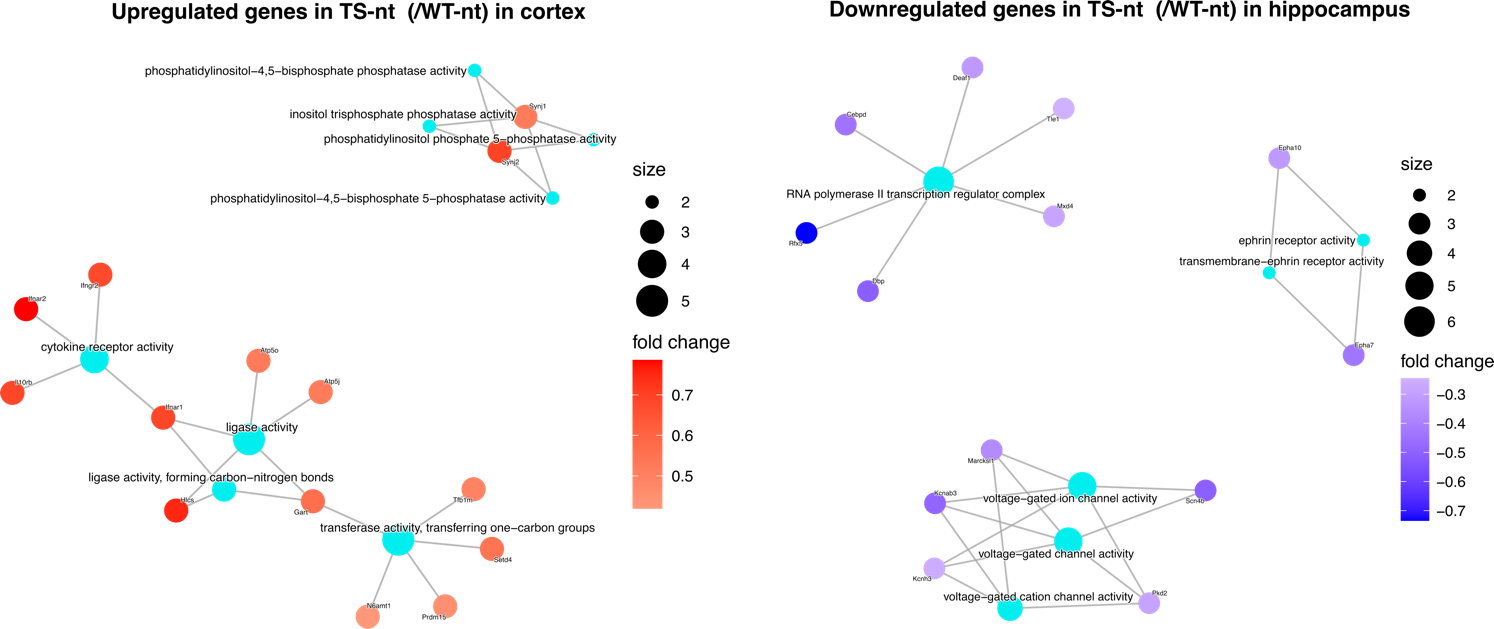
**

**Figure S3. Functional enrichment analysis of DE genes in genotype effect comparisons (TS-nt vs. WT-nt).** Cnetplot showing gene linkages and biological concepts identified as GO terms represented as networks. Enriched functions and genes are listed in Supplementary Table S1.

**
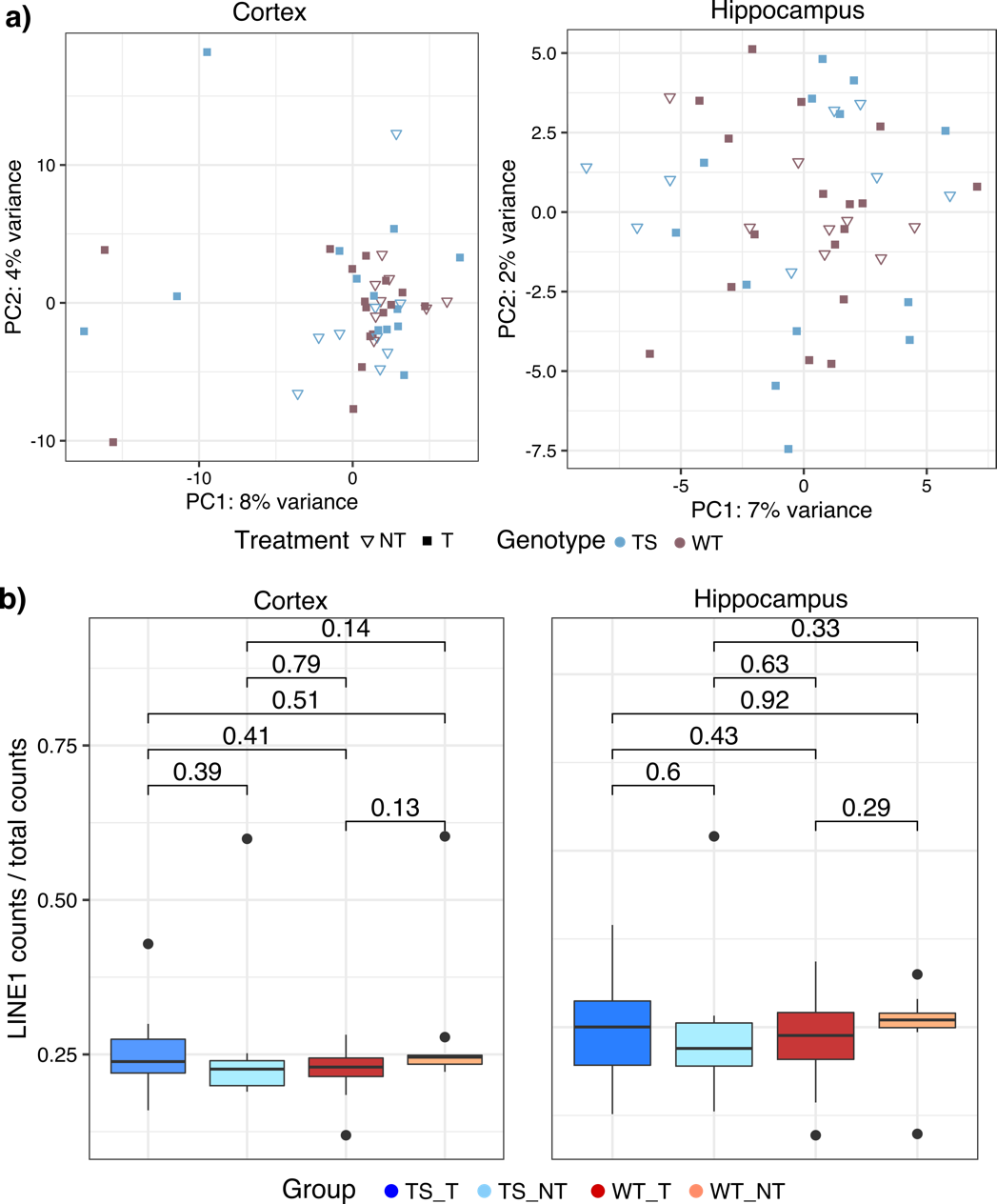
**

**Figure S4. LINE1 expression profiling in cortex and hippocampus. a)** Principal component analysis using LINE1 expression (rlog normalization) by RNA sequencing. **b)** Boxplots displaying proportion of LINE1 counts (all elements merged) over total RNA counts.


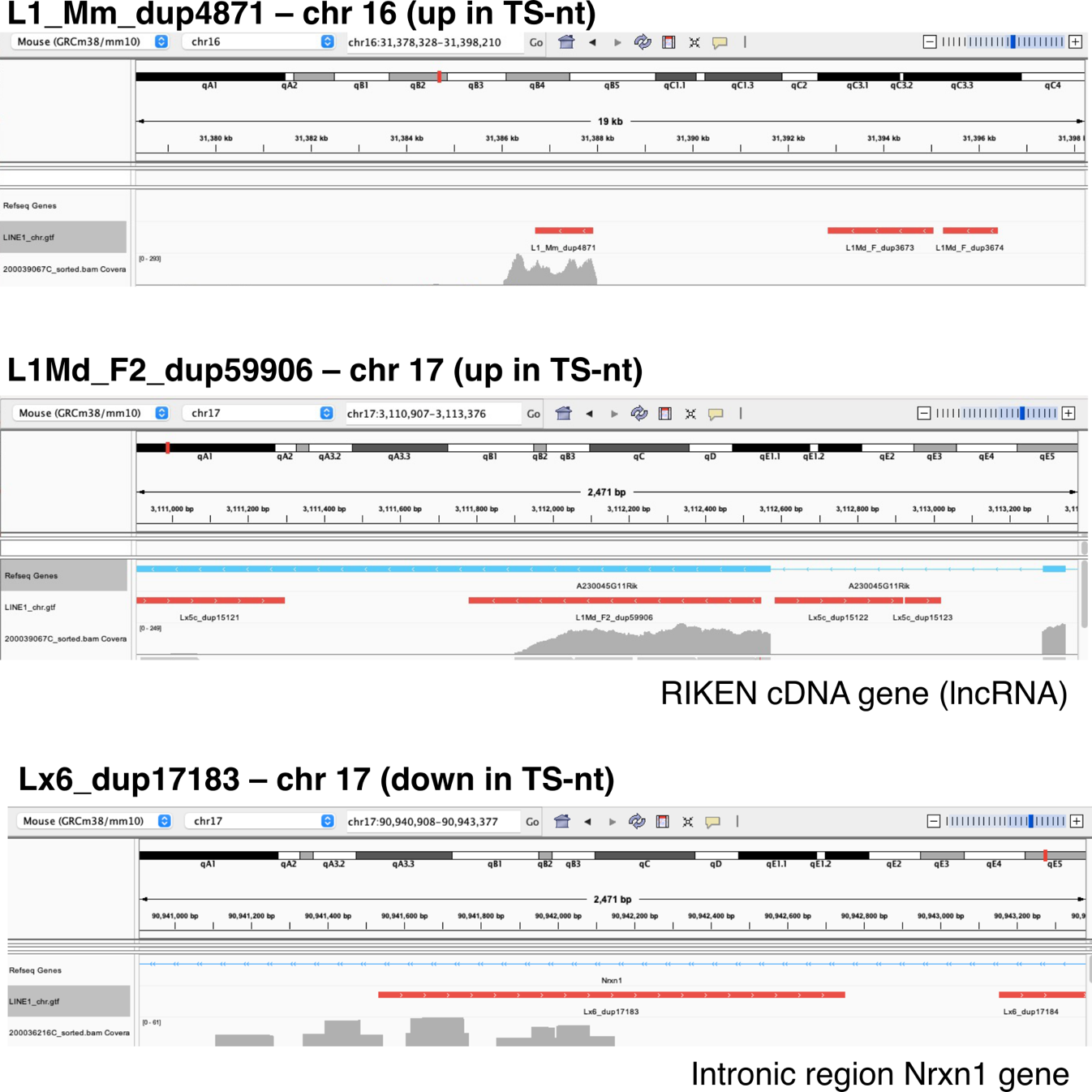


**Figure S5. Chromosome location (Mmu 16 and 17) of differentially expressed LINE1s in the genotype effect (TS-nt vs. WT-nt).** IGV overview showing the sequence alignment (gray), reference gene (light blue) and LINE1 (red) annotation in the corresponding chromosome region in the cortex and hippocampus.

**
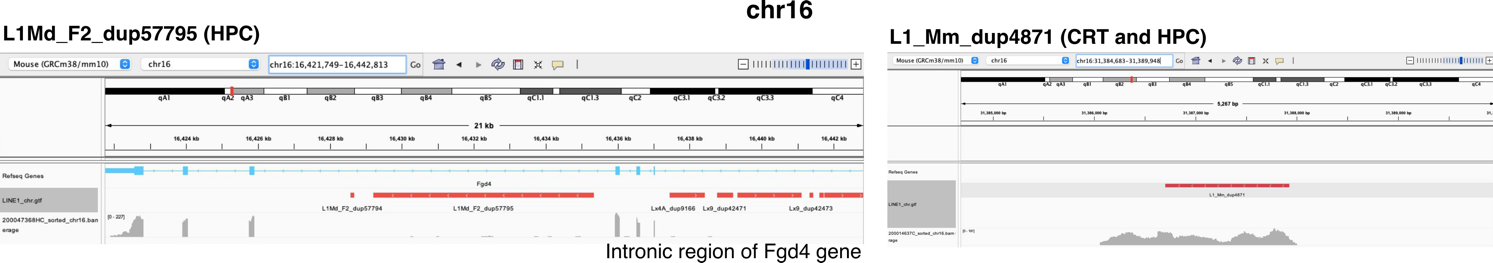
**

**
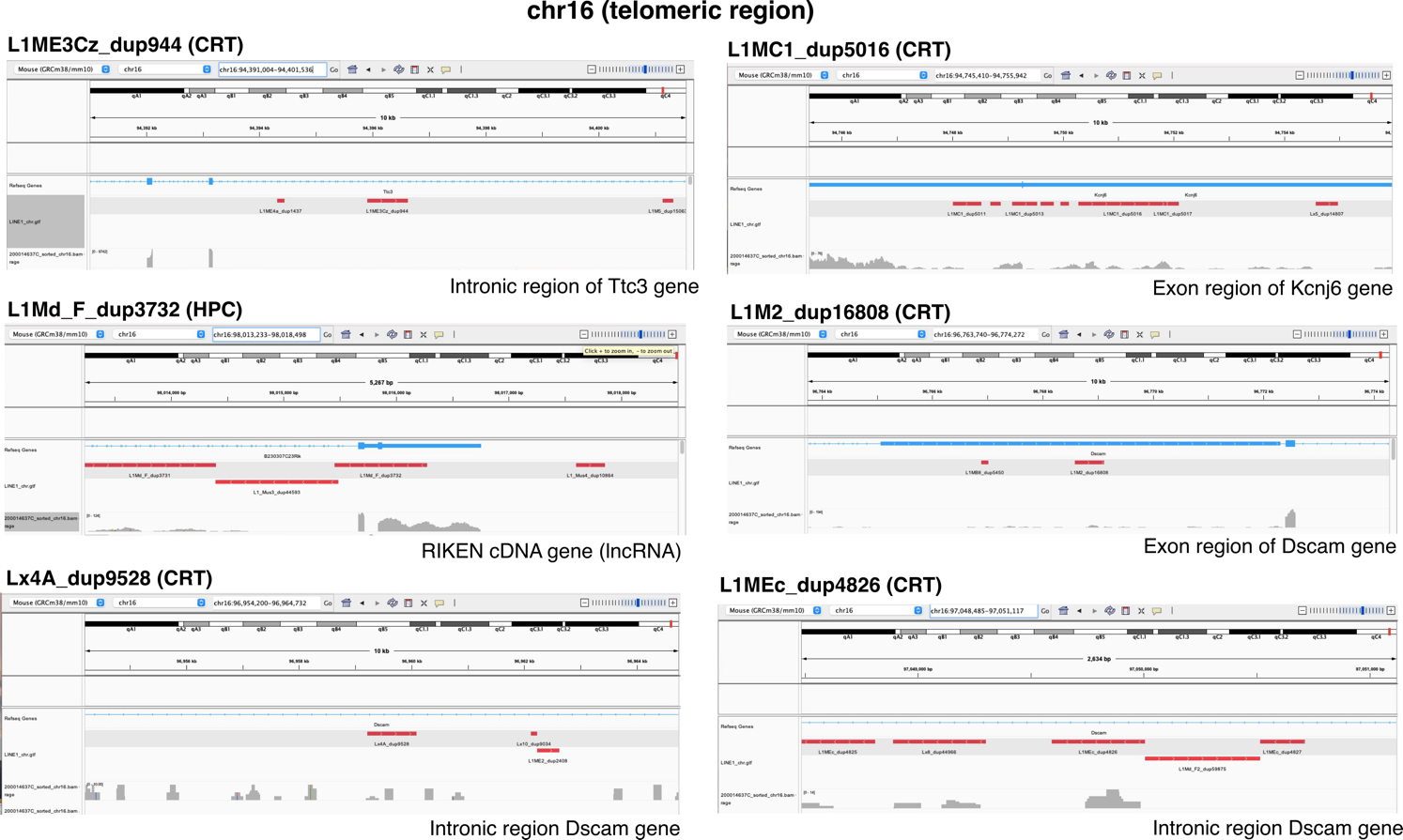
**

**
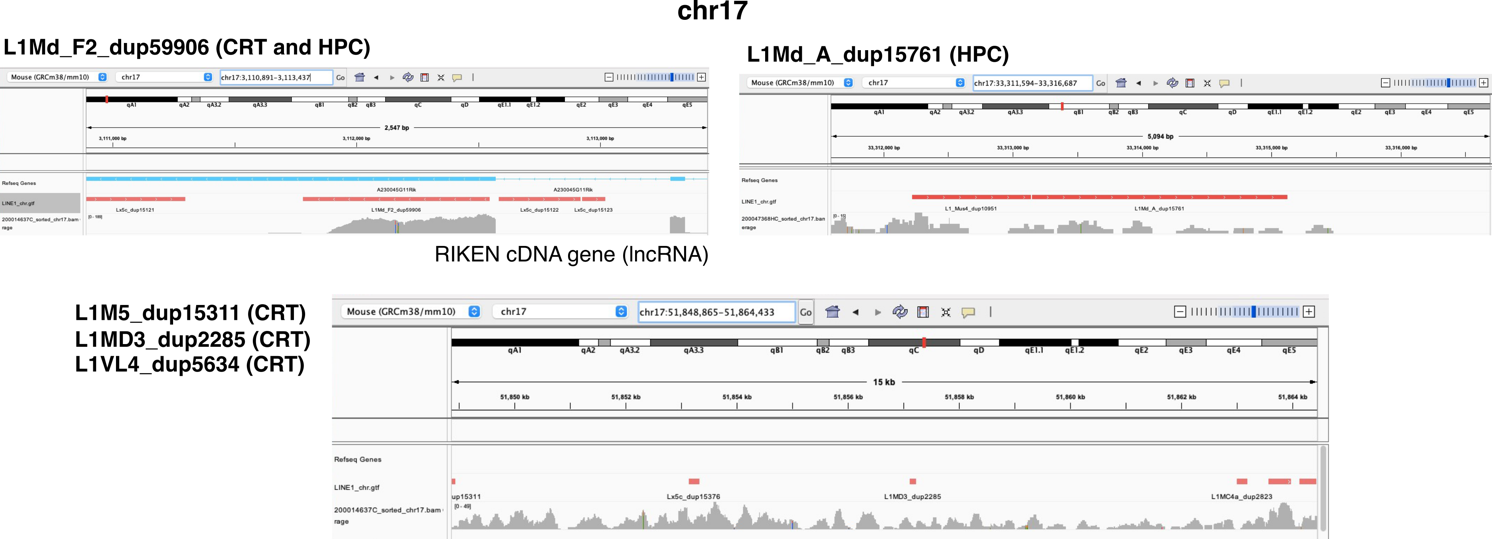
**

**Figure S6. Chromosome location (MMU 16 and 17) of LINE1s in the comparison TS-t vs. WT-nt.** IGV overview showing the sequence alignment (gray), reference gene (light blue) and LINE1 (red) annotation in the corresponding chromosome region in the cortex and hippocampus.


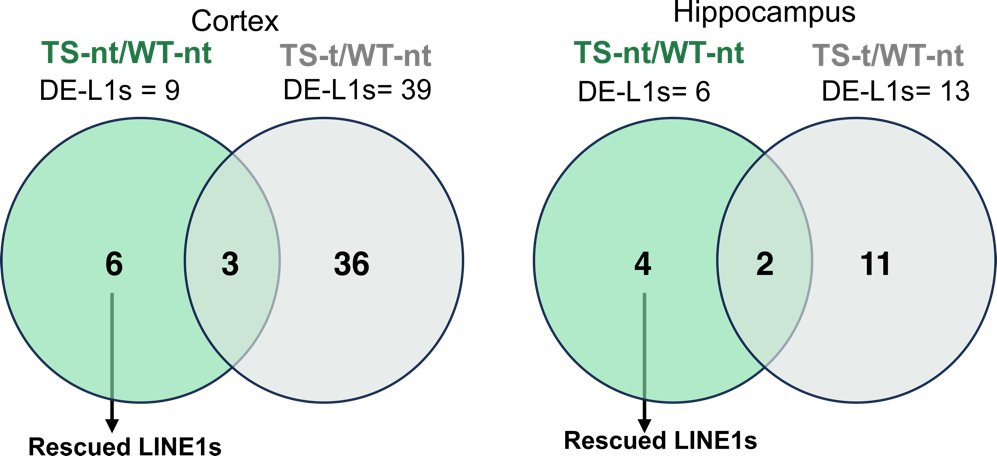


**Figure S7. Rescued LINE1s after lamivudine treatment in the cortex and hippocampus.** Venn diagrams showing LINE1s that were restored (rescued) after treatment with lamivudine (those altered by genotype that are not present in treated mice, and that correct their levels to WTs).

**
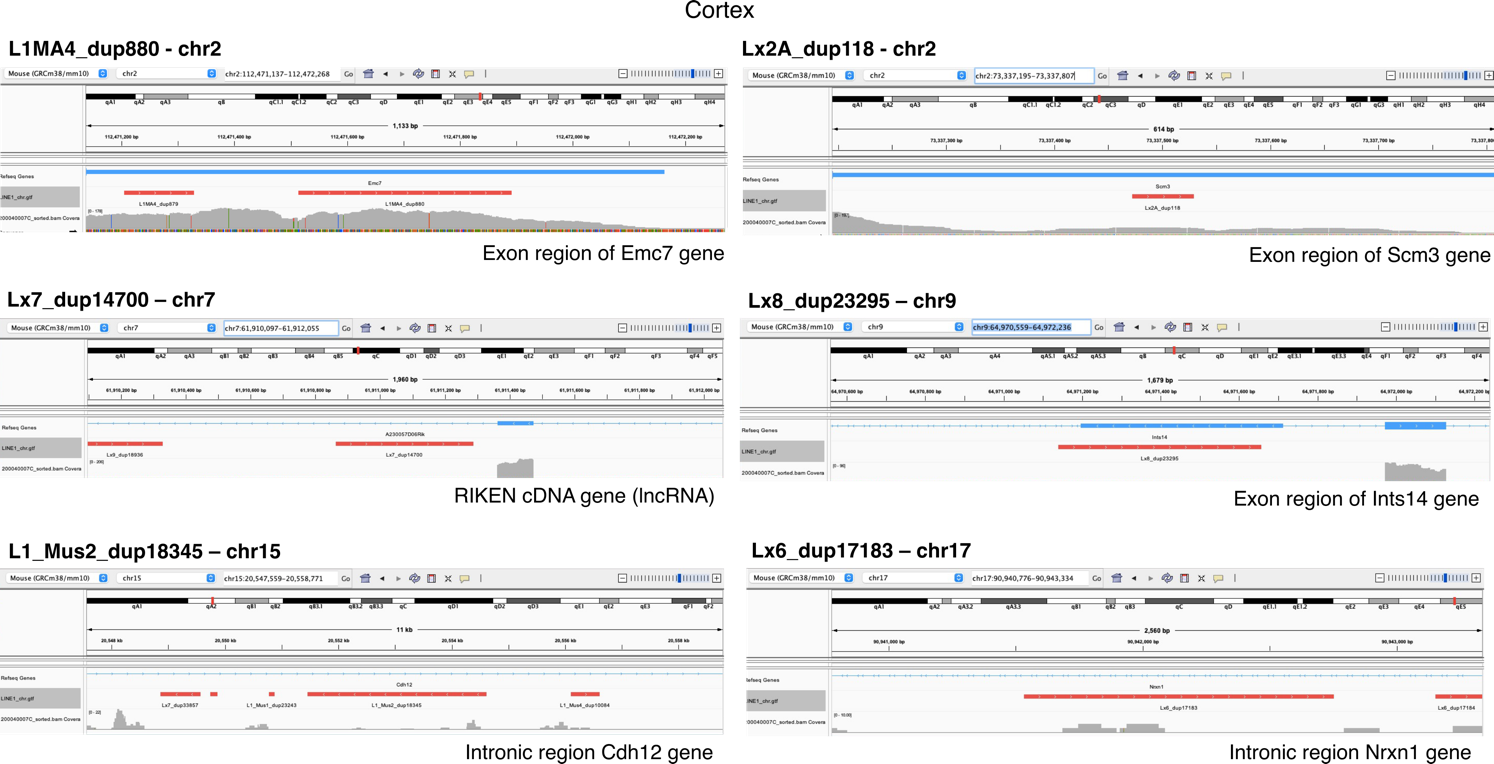
**


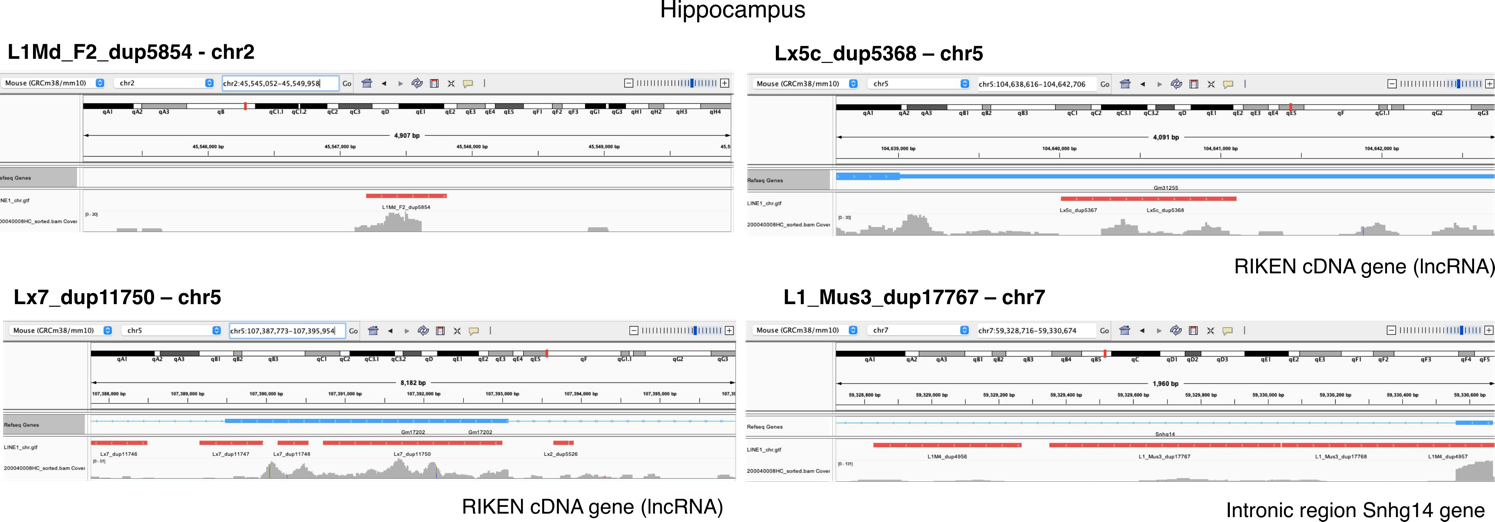


**Figure S8. Chromosome location of all LINE1s rescued after treatment with lamivudine.** IGV overview showing the sequence alignment (gray), reference gene (light blue) and LINE1 (red) annotation in the corresponding chromosome region in the cortex and hippocampus.

**Figure S9. Correlation analysis between senescence markers and rescued LINE1s.** Spearman rank correlations between senescence markers and rescued LINE1s (L1s) based on normalized expression levels (rlog). Color and size of the square represent the magnitude of the correlation. Significance is indicated by white asterisks (*p < 0.05; **p < 0.01; ***p < 0.001 after Benjamini–Hochberg adjustment for multiple comparisons).

**Figure S10. Correlation plot between senescence markers and DE LINE1s in TS-t/WT-nt.** Spearman correlations between senescence markers and DE LINE1s (L1s) in TS-t (vs. WT-nt) mice. Correlation analysis is based on normalized expression levels (rlog). Color and size of the square represent the magnitude of the correlation. Significance is indicated by white asterisks (*p < 0.05; **p < 0.01; ***p < 0.001 after Benjamini–Hochberg adjustment for multiple comparisons).

**
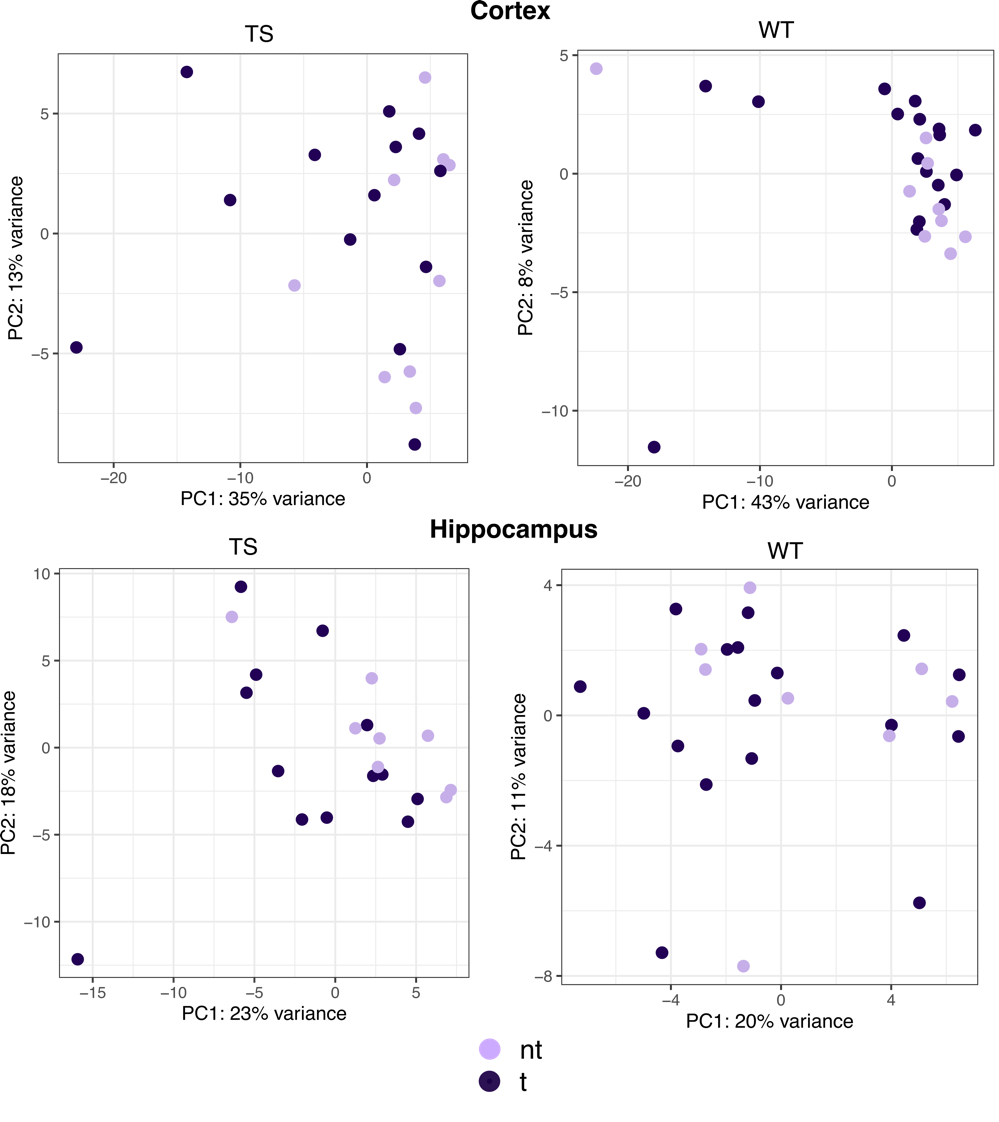
**

**Figure S11. Principal component analysis (PCA) by genotype and tissue.** PCA displaying sample clustering based on gene expression of mice groups treated (t) and not treated (nt) with lamivudine.


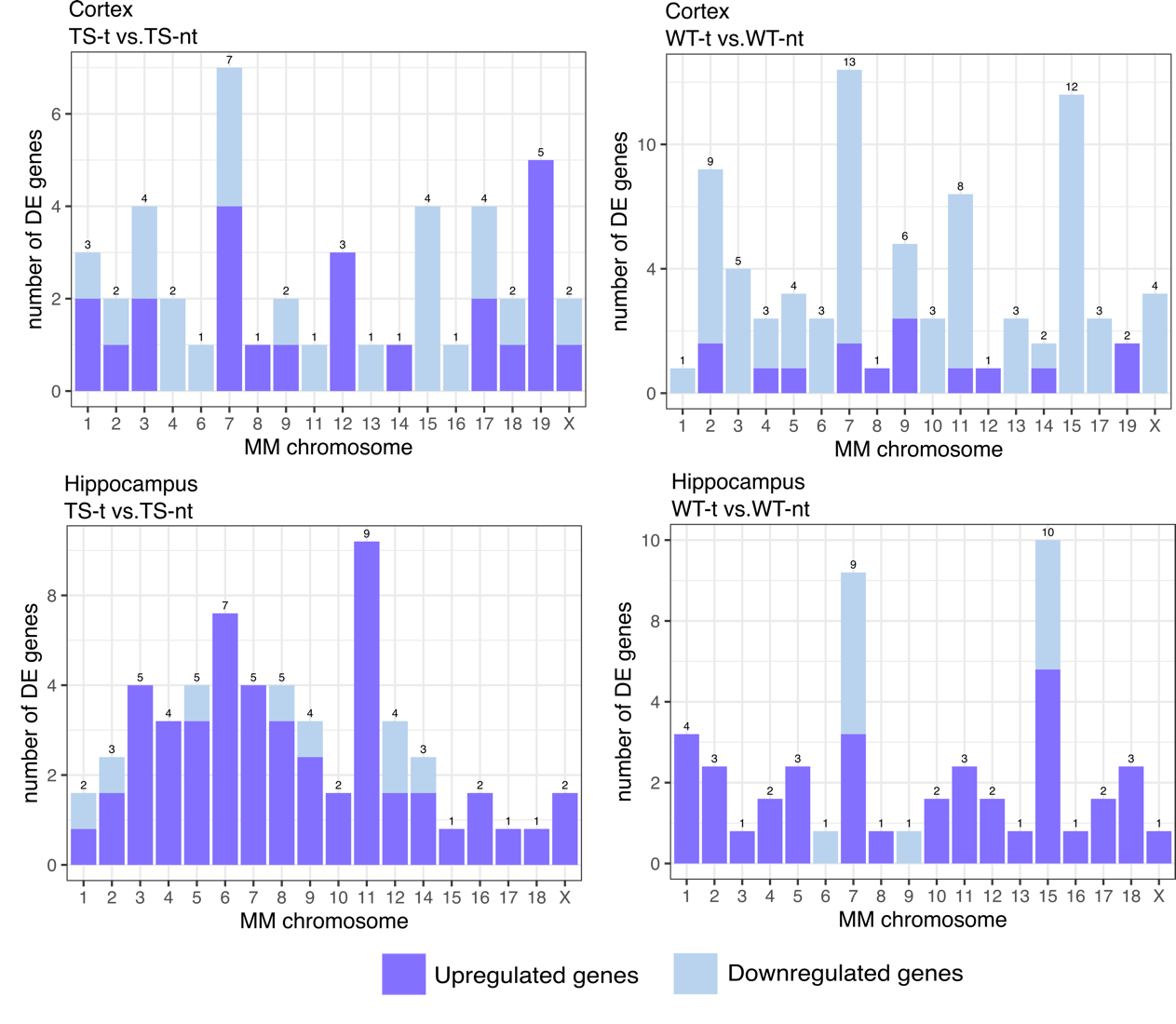


**Figure S12. Chromosome mapping of DE genes in comparison pairs for treatment effect assessment.** Barplot for the number of DE genes per mouse chromosomes mapping to their human orthologs in the cortex and hippocampus.


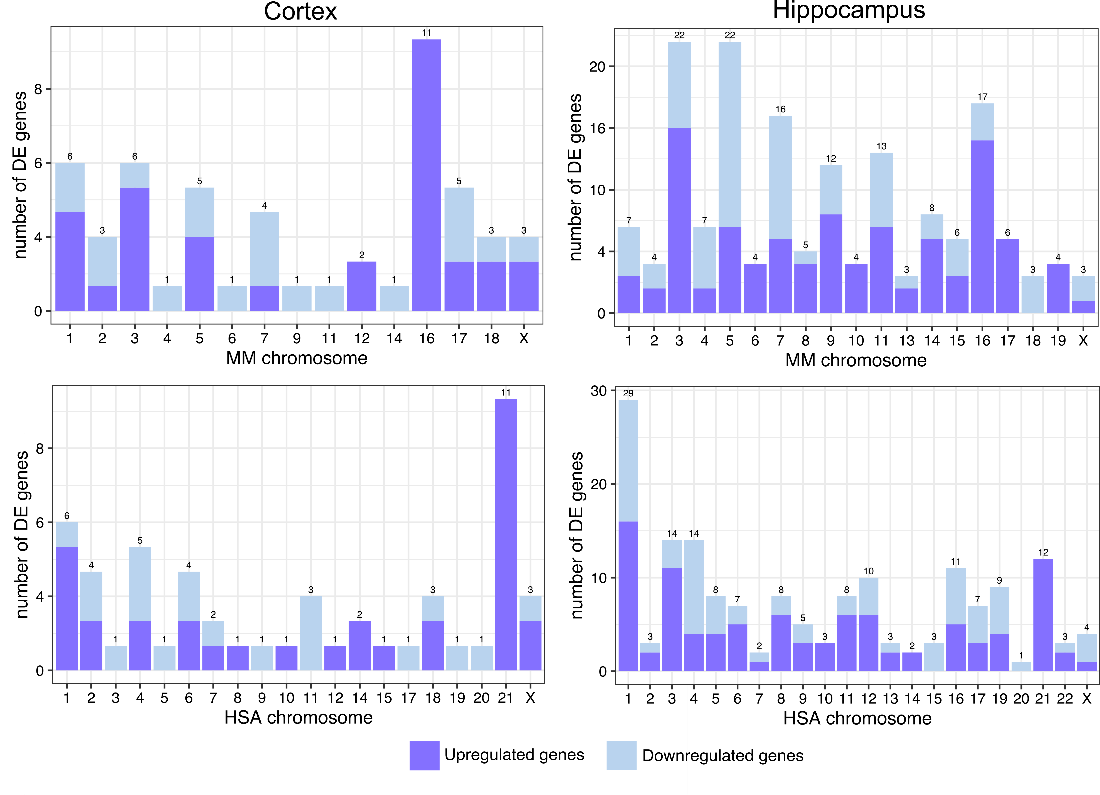


**Figure S13.** Barplot for the number of rescued genes per mouse chromosomes mapping to their human orthologs identified in cortex and hippocampus.

**
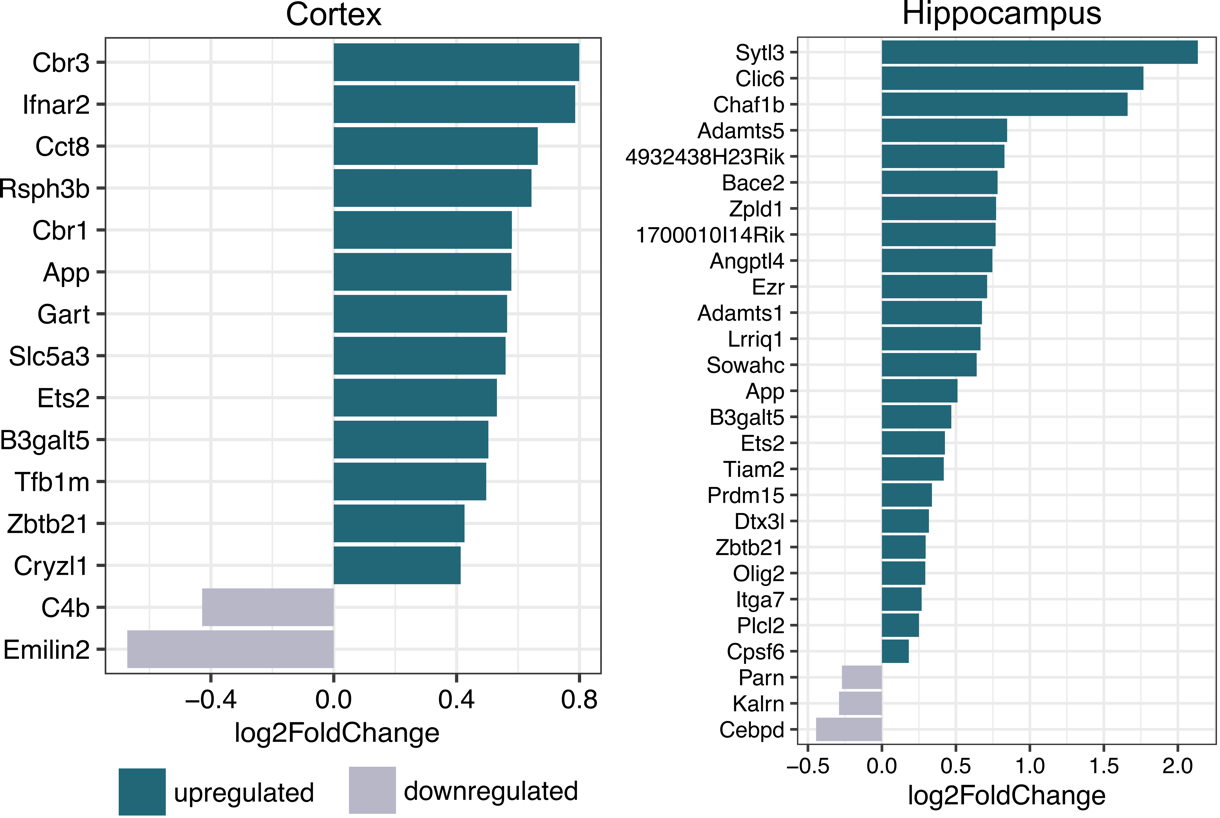
**

**Figure S14. Differential expression of rescued genes located in MMU10, 16 and 17.** Barplots showing rescued genes expression in the cortex and the hippocampus sorted by log2 fold change.


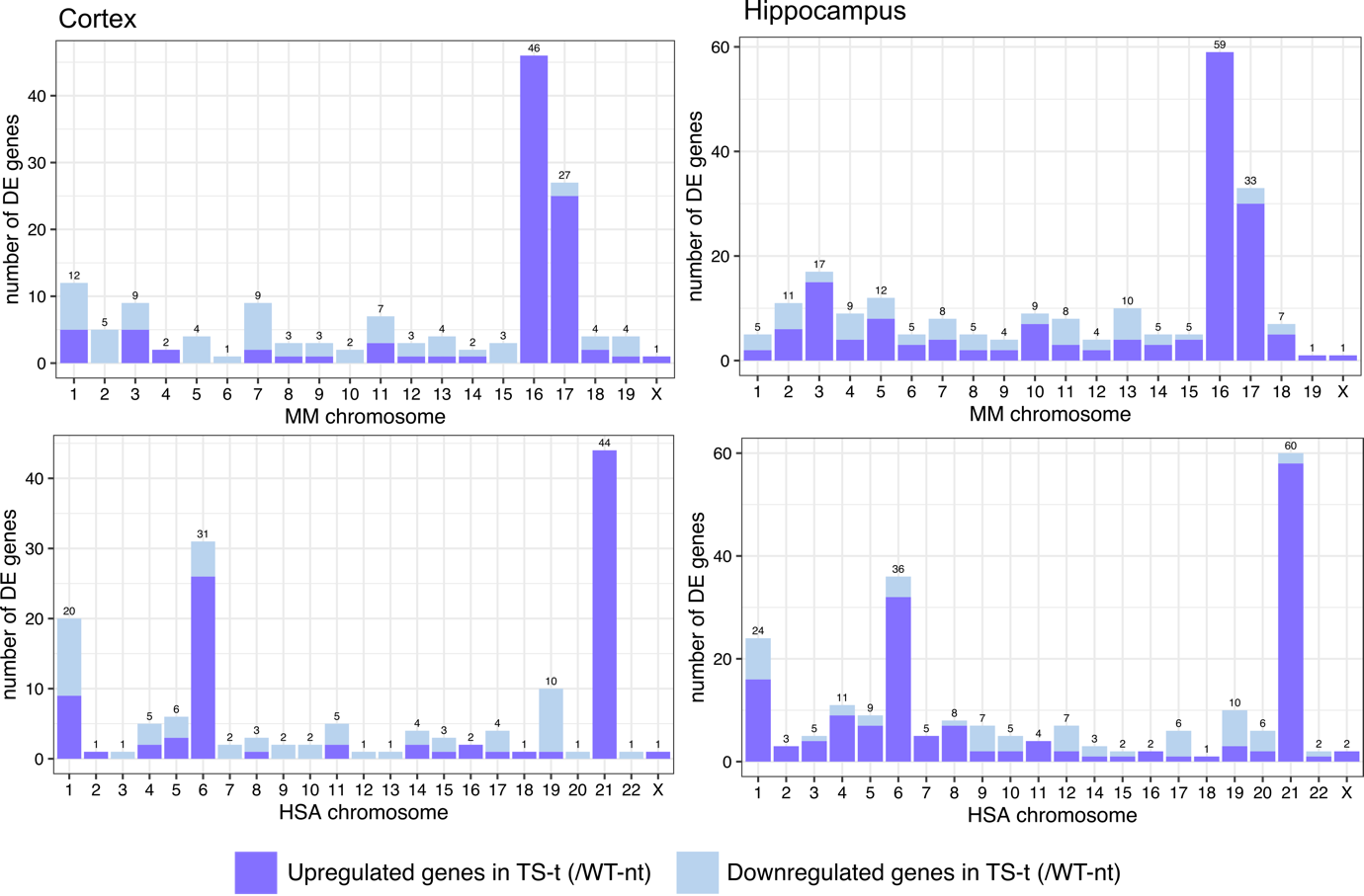


**Figure S15. Chromosome mapping of DE genes in the comparison between TS-t vs. WT-nt mice.** Barplot for the number of DE genes per mouse chromosomes mapping to their human orthologs in the cortex and hippocampus.


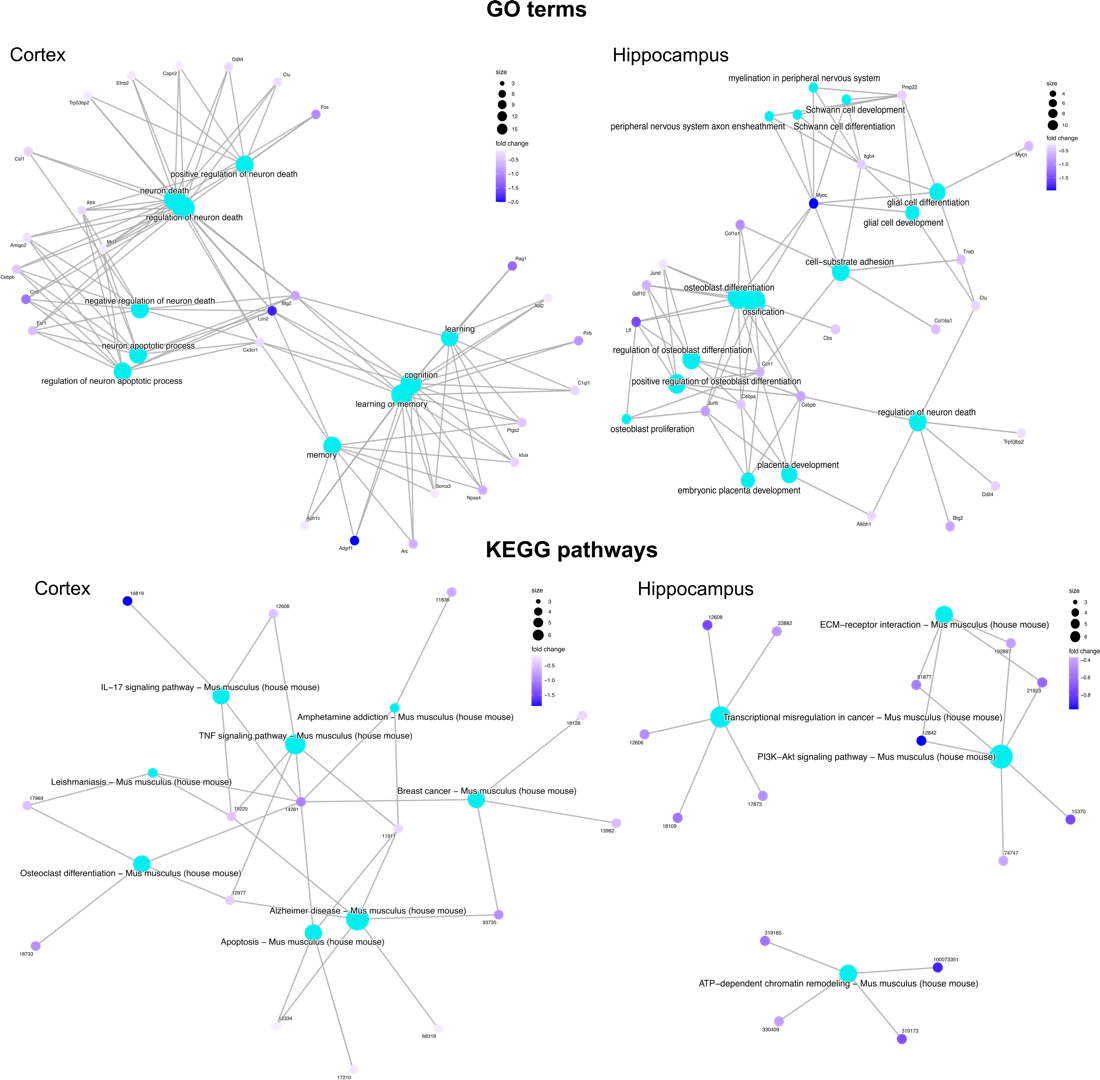


**Figure S16. Functional enrichment analysis of downregulated genes in TS-t vs. WT-nt mice.** Cnetplot showing linkages of downregulated genes and biological concepts identified as GO terms and KEGG pathways, represented as networks. Enriched functions and related genes are listed in Supplementary Table S5.


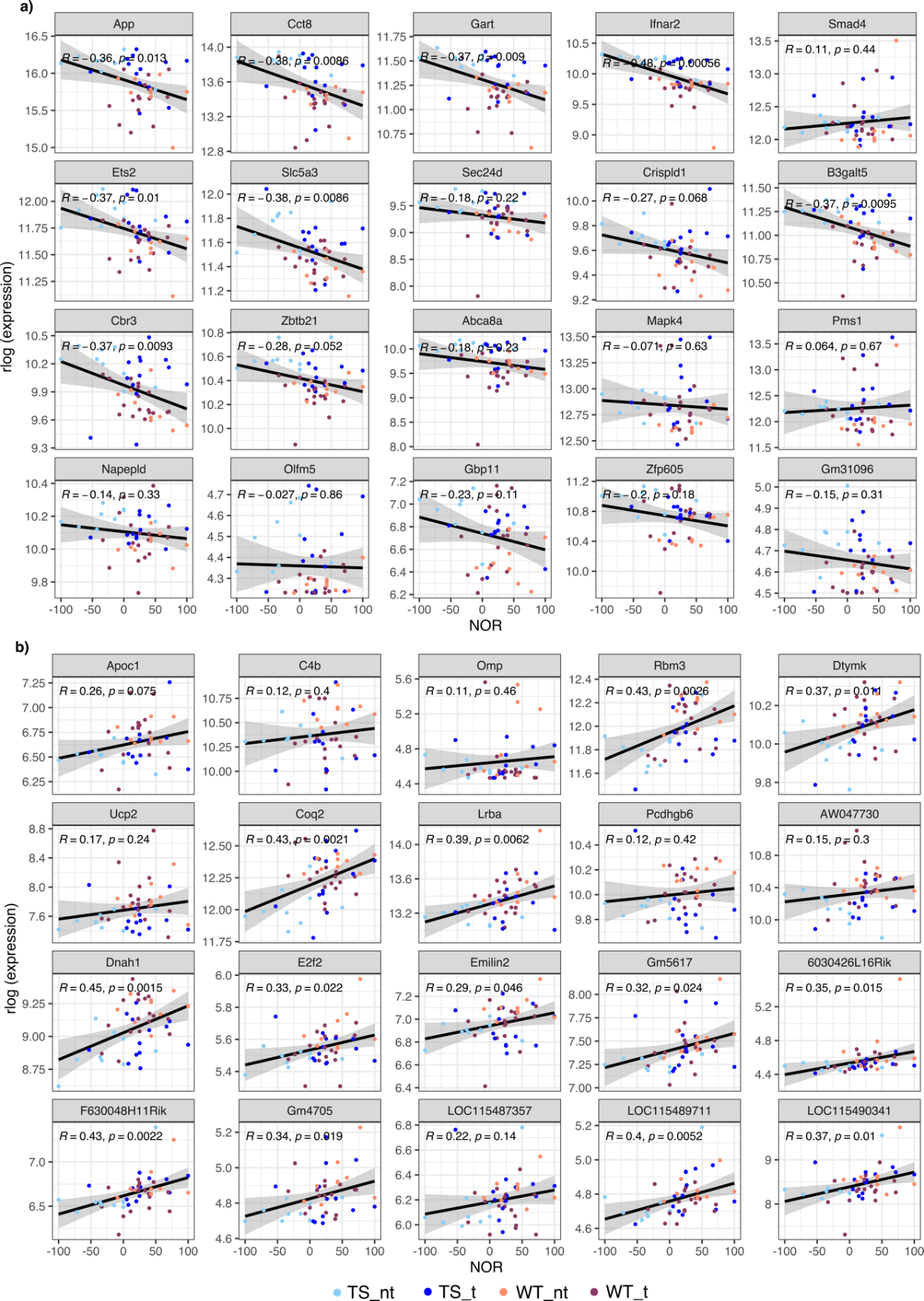


**Figure S17. Correlation between rescued genes and discrimination index for all mice groups in the cortex.** Scatterplot showing correlation between discrimination index at 4 months (NOR) and normalized expression (rlog normalized counts) of rescued genes across all mouse chromosomes. Top 20 most discriminant a) upregulated and b) downregulated rescued genes were represented (genes were sorted by adjusted p-value significance and fold change).


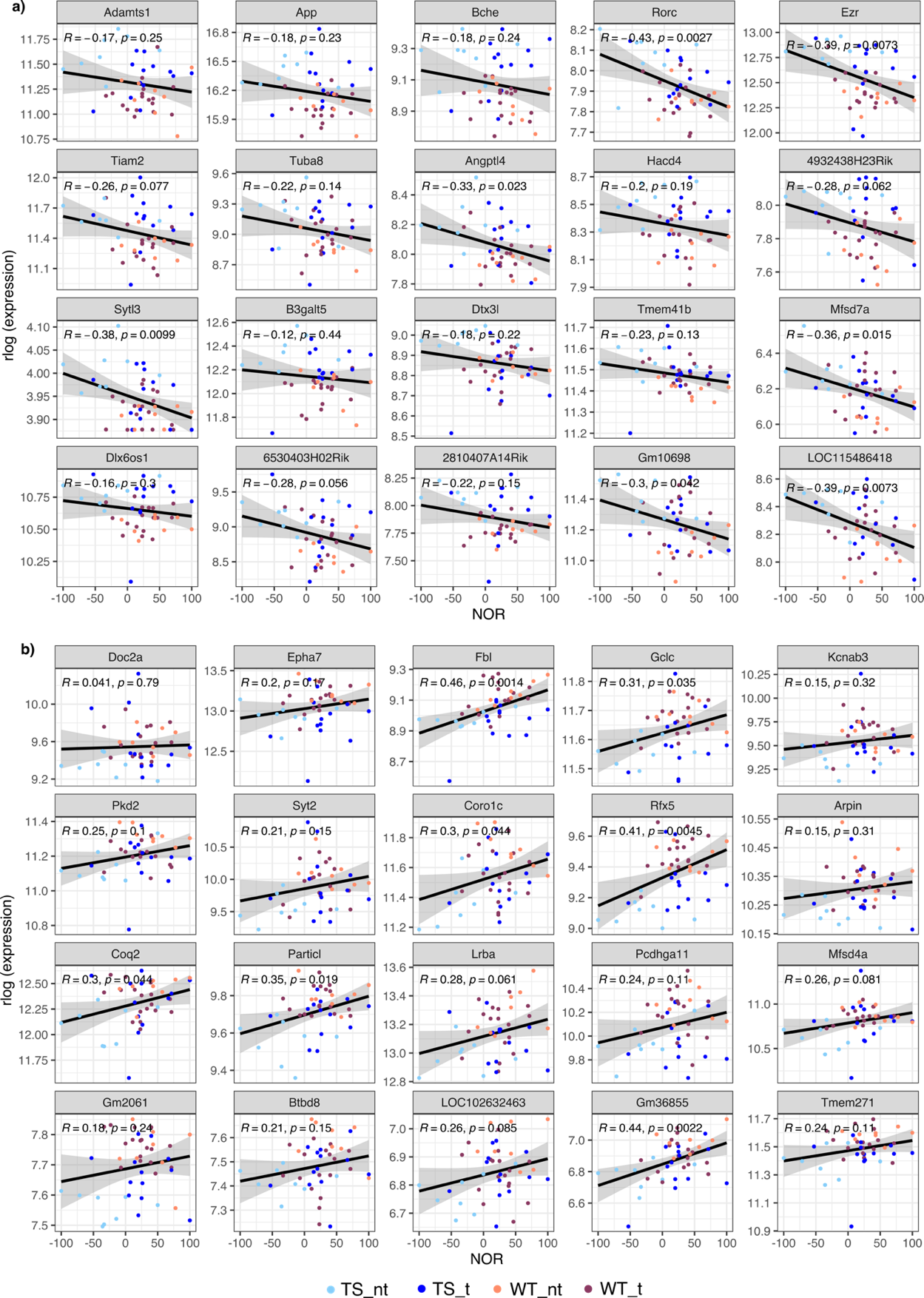


**Figure S18. Correlation between rescued genes and discrimination index for all mice groups in the hippocampus.** Scatterplot showing correlation between discrimination index at 4 months (NOR) and normalized expression (rlog normalized counts) of rescued genes across all mouse chromosomes. Top 20 most discriminant a) upregulated and b) downregulated rescued genes were represented (genes were sorted by adjusted p-value significance and fold change).


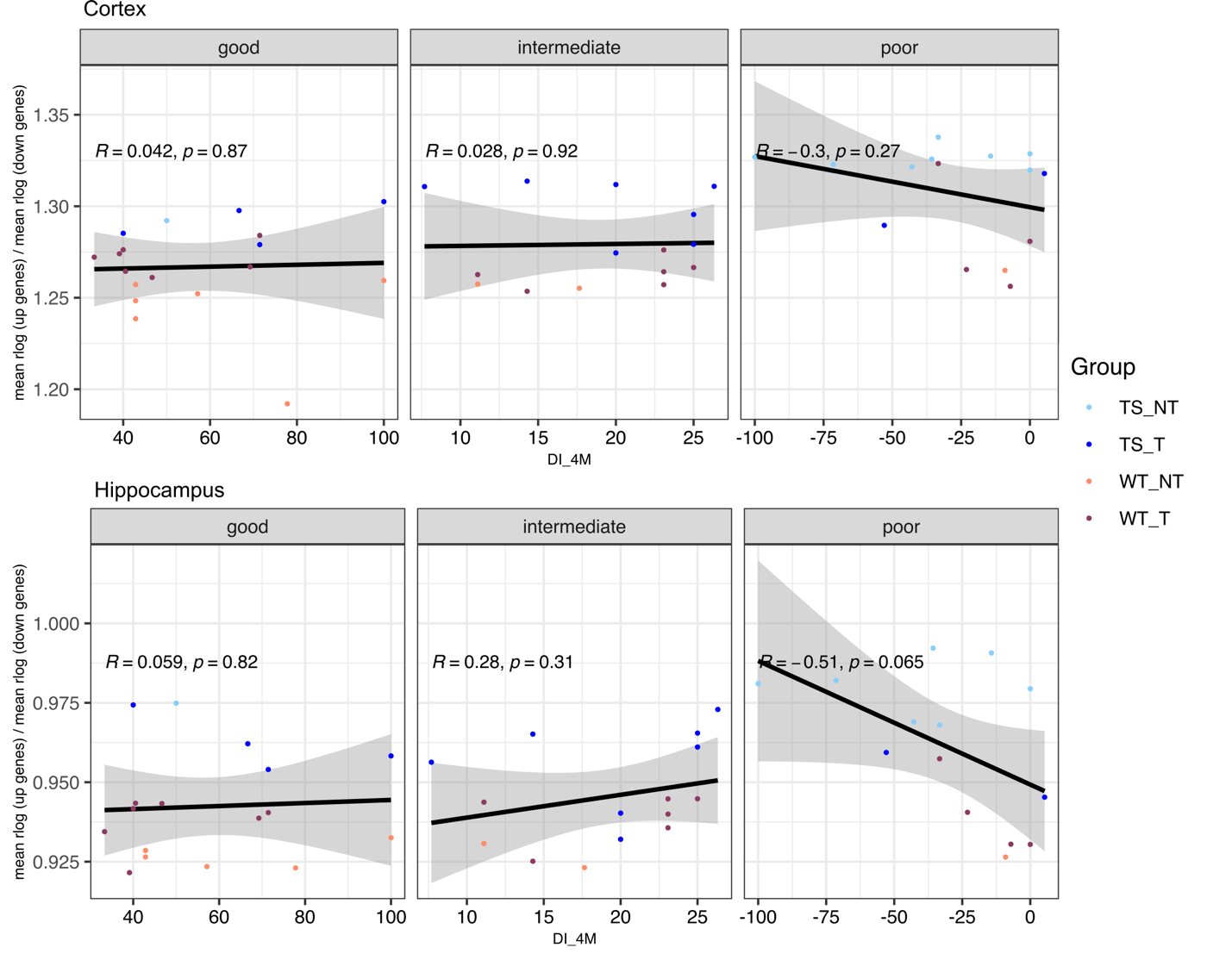


**Figure** **S19**. **Correlations between rescued gene ratio and discrimination index in mice stratified by learning performance.** Scatterplot showing Spearman’s correlations between discrimination index after 4 months of treatment (DI_4M) and rescued gene ratio (mean of normalized upregulated rescued gene expression / mean of normalized downregulated rescued gene expression, across all mouse chromosomes) in cortex and hippocampus of mice stratified by learning performance (DI_4M group mean ± 3 SEM).
